# The Plant Cell Wall: From Structural Diversity and Biosynthesis to Integrity Signaling and Physiological Functions

**DOI:** 10.3390/plants15182835

**Published:** 2026-09-16

**Authors:** Yamei Zhuang, Shujuan Zhang, Mingcheng Zhang, Qingqi Fan, Cheng Liu, Xianfeng Tang, Gongke Zhou, Guang Qi

**Affiliations:** 1 Crop Research Institute, Shandong Academy of Agricultural Sciences, Jinan 252100, China; 18954218411@163.com (Y.Z.); zsjhappy@163.com (S.Z.); zmc163mail@163.com (M.Z.); fanqingqi@163.com (Q.F.); lch6688407@163.com (C.L.); 2 National Key Laboratory of Wheat Improvement, Jinan 252100, China; 3 College of Landscape Architecture and Forestry, Qingdao Agricultural University, Qingdao 266109, China; tangxf@qau.edu.cn; 4 College of Resources and Environment, Qingdao Agricultural University, Qingdao 266109, China; zhougk@qau.edu.cn

**Keywords:** cell wall, biosynthesis, assembly, cell wall integrity, physiological function

## Abstract

Plant cell walls are dynamic structural matrices that provide mechanical support while regulating growth, transport, environmental sensing, and defense. Their properties emerge from the coordinated biosynthesis, deposition, assembly, and remodeling of cellulose, hemicelluloses, pectins, lignin, and specialized wall polymers, yet how wall architecture is coupled to integrity surveillance and whole-plant physiology remains incompletely understood. Here, we synthesize current knowledge of plant cell wall classification, major polymer biosynthesis and assembly, cell wall integrity signaling, and physiological functions. We compare primary, secondary, and specialized walls and discuss how differences in their composition and organization confer distinct mechanical and functional properties. We then examine how wall-derived oligosaccharides, apoplastic peptides, receptor-like kinases, Ca^2+^ signaling, reactive oxygen species, cytoskeletal dynamics, and hormonal crosstalk translate wall perturbations into adaptive responses. Viewed in an integrated physiological context, wall composition, architecture, and remodeling shape anisotropic growth, morphogenesis, tissue repair, xylem hydraulics, ion homeostasis, abiotic stress adaptation, and pathogen defense. Collectively, current evidence supports a view of the cell wall as an active regulatory interface rather than a passive scaffold. Finally, we highlight unresolved questions concerning mechanochemical sensing, spatial heterogeneity, and growth–defense trade-offs, and discuss how advances in spatial omics, live-cell imaging, biomechanics, genome editing, and computational modeling may help resolve how wall properties are regulated across cells, tissues, and environmental contexts.

## 1. Introduction

The plant cell wall is a structurally complex and compositionally diverse integral component of plant cells that shapes their morphology, mechanics, and function. Its architecture is built primarily from cellulose microfibrils embedded in matrices of hemicelluloses, pectins, structural proteins, and, in many secondary walls, lignin [1]. As one of the most abundant renewable resources on Earth, plant cell walls constitute the major fraction of lignocellulosic biomass and provide essential raw materials for pulping and papermaking, ruminant feed, and biofuel production [2]. The latest FAO statistics for 2024 (FAOSTAT, accessed in August 2026) indicate that global production of major plant-cell-wall-derived commodities reached 1.96 billion m^3^ of industrial roundwood, 393 million m^3^ of wood-based panels, 189 million tonnes of wood pulp, and 48 million tonnes of wood pellets, with the international export value of wood and paper products amounting to US$486 billion as an economic proxy (https://doi.org/10.4060/cd8005en). Beyond their economic importance, cell walls are central to plant biology. Once regarded mainly as passive structural barriers, they are now recognized as dynamic regulatory interfaces that integrate mechanical support, morphogenesis, water and nutrient transport, environmental sensing, and defense responses. Accordingly, understanding wall structure and remodeling is critical not only for explaining plant growth, development, and stress adaptation, but also for improving crop traits such as lodging resistance, vascular function, stress tolerance, disease resistance, and biomass processing efficiency [3].

Recent advances in genetics, cell biology, imaging, biomechanics, glycomics, and omics-based approaches have greatly expanded our understanding of cell wall composition, biosynthesis, assembly, and function. However, major gaps remain in explaining how local changes in cell wall properties and integrity are sensed and translated into developmental, metabolic, and stress-related responses [4,5]. In this review, we summarize current knowledge of plant cell wall classification, major wall components, polymer biosynthesis, wall assembly, cell wall integrity signaling, and wall remodeling dynamics. We further discuss how these processes contribute to plant growth, development, abiotic stress adaptation, ion homeostasis, and plant–microbe interactions. By integrating structural, biochemical, developmental, and signaling perspectives, this review highlights the plant cell wall as a dynamic regulatory system essential for plant survival, adaptation, and agricultural improvement.

## 2. Classification of Plant Cell Walls

Plant cell walls can be classified into primary and secondary walls based on developmental timing, chemical composition, architecture, and function [6]. While the distinction between primary and secondary walls is foundational due to differences in composition and mechanics, cell walls remain dynamic, continuously remodeled in response to developmental and environmental signals. In addition to these, cells frequently develop specialized walls via modifications such as cutinization, suberization, or callose deposition. Such specialized structures underpin a variety of physiological functions, including protection, transport, reproduction, nutrient exchange, and adaptation to environmental stress [6].

### 2.1. Primary Cell Walls

Primary cell walls are formed outside the plasma membrane during cytokinesis and are continuously remodeled throughout cell expansion [1,6]. Based on their composition, they are generally divided into two major types that differ in wall extensibility, digestibility, pathogen interactions, and mechanical properties [6]. Type I walls, found in most dicots, non-commelinid monocots, and gymnosperms, are rich in pectins and xyloglucans, with current models suggesting that xyloglucans contribute to wall mechanics mainly through localized cellulose–hemicellulose load-bearing “hotspots” rather than extensive cellulose tethering. In contrast, Type II walls, typical of grasses and other commelinid monocots, contain less pectin and xyloglucan but are enriched in glucuronoarabinoxylans and mixed-linkage glucans [6]. Beyond their structural role, primary walls also function as dynamic signaling interfaces, where changes in wall integrity, mechanical stress, or polymer organization are sensed by plasma membrane-localized receptors and transduced through Ca^2+^, reactive oxygen species, and hormone signaling pathways, thereby promoting transcriptional reprogramming and compensatory wall remodeling in response to developmental and environmental cues [7].

### 2.2. Secondary Cell Walls

Secondary cell walls are deposited after cell expansion has largely ceased, forming structures that are thicker, stiffer, and more specialized than primary walls. They are characteristic of cells involved in mechanical support and long-distance water transport, such as xylem vessels, tracheids, and many fibers. In tracheary elements, they remain functional even after programmed cell death removes the protoplast [1]. These walls are typically rich in cellulose and hemicelluloses, often with substantial lignin but relatively little pectin. Their hemicellulose composition varies phylogenetically, with xylans predominating in angiosperms and glucomannans being more abundant in gymnosperms [1,8]. Together with cellulose microfibrils, these polymers help define wall architecture, while lignification in vascular tissues provides compressive strength, hydrophobicity, and resistance to microbial degradation. However, lignification is not universal; for example, cotton fibers develop thick secondary walls that are highly enriched in cellulose but contain little or no lignin.

**Figure 1:**
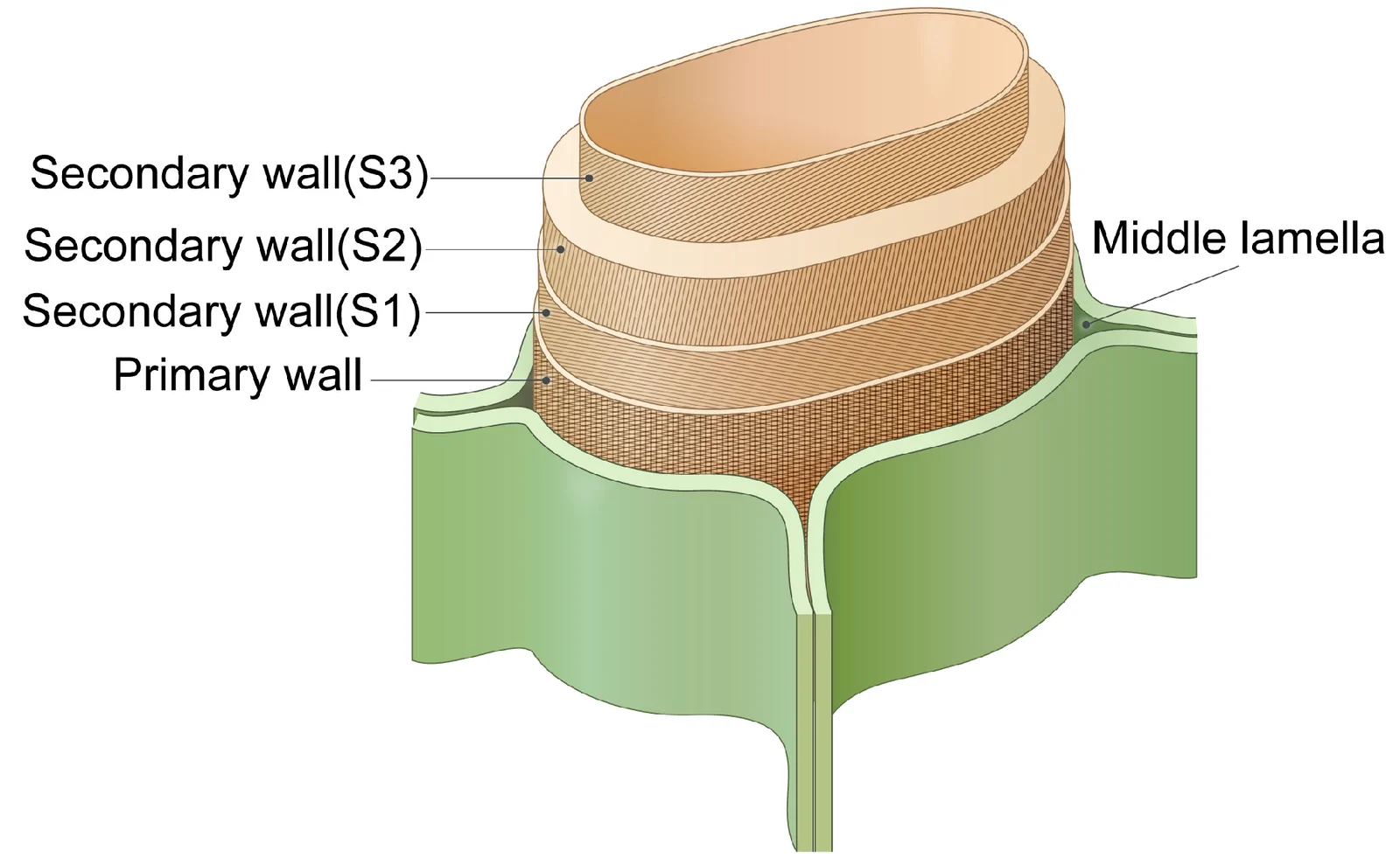
Schematic architecture of a lignified plant cell wall. The diagram illustrates the spatial organization of major wall layers, including the middle lamella, primary wall, and the three sublayers (S1, S2, S3) of the secondary wall. The relative thickness and position of each layer are depicted, highlighting the structural complexity of the lignified cell wall.

In many secondary walls, particularly in wood fibers and tracheary cells, the wall is classically divided into S1, S2, and S3 layers, which differ in thickness, composition, and cellulose microfibril angle [8]. The S2 layer contributes the most to mechanical strength, and its microfibril orientation strongly influences wall stiffness and flexibility (Figure 1). Secondary wall biosynthesis is tightly controlled by developmental programs that coordinate the production and deposition of cellulose, hemicelluloses, and lignin precursors [9]. Deposition is also spatially patterned according to cell function: in xylem elements, annular, spiral, reticulate, or pitted thickenings balance mechanical support with hydraulic performance, while pits permit lateral water movement between adjacent conduits. As a major component of lignocellulosic biomass, secondary walls are also of broad ecological and economic importance, supporting industries such as timber, paper, textiles, forage, and bioenergy while also presenting challenges for biomass processing.

### 2.3. Specialized Cell Walls

In addition to primary and secondary walls, plants also produce specialized cell wall structures adapted to particular physiological functions through unusual polymers, distinctive deposition patterns, and targeted chemical modifications [10]. A major category comprises protective extracellular barriers. Cutin forms the structural framework of the epidermal cuticle and, together with cuticular waxes, reduces non-stomatal water loss, limits pathogen entry, and protects aerial tissues from environmental stress [11]. Likewise, suberin is deposited in the endodermis, periderm, and wound-associated tissues, where it restricts the uncontrolled movement of water, ions, and pathogens; in roots, suberin lamellae act together with the lignin-rich Casparian strip to form an endodermal diffusion barrier that regulates radial transport and selective nutrient uptake [10,11]. Another widespread specialization is callose, a rapidly deposited β-1,3-glucan that accumulates at plasmodesmata, sieve plates, developing cell plates, pollen tubes, and wound or infection sites, where it modulates intercellular transport, reinforces local wall regions, and contributes to defense responses [12]. In some lineages, especially grasses, silica deposition provides additional wall reinforcement and enhances resistance to environmental and biotic stresses [13].

Specialized walls also support solute exchange, hydration control, reproduction, and developmental remodeling. Transfer cells develop extensive wall ingrowths that increase plasma membrane surface area and thereby enhance transport efficiency in high-flux tissues [7]. In many species, seed coat epidermal cells secrete pectin-rich mucilage that expands upon hydration, influencing water uptake, soil adhesion, and germination [8]. Pollen walls represent another highly specialized extracellular structure, consisting of an inner intine and an outer exine reinforced with sporopollenin, which protects pollen from desiccation and microbial attack and contributes to pollen-stigma interactions; after germination, the pollen tube wall undergoes polarized remodeling, with an extensible apex and progressively reinforced subapical regions that help maintain tube integrity during fertilization [12,14]. Other examples include guard cell walls, whose anisotropic reinforcement converts turgor changes into stomatal movement, root hair walls that sustain tip growth through localized remodeling, and abscission-zone walls that undergo loosening and middle lamella dissolution during organ separation [15,16,17]. In addition, the cell corner, a specialized wall domain located at the junction between adjacent cells, exhibits compositional heterogeneity among cell types and across developmental stages, consistent with its roles in cell–cell adhesion and localized mechanical reinforcement [6]. Together, these examples illustrate the remarkable functional versatility of plant cell walls.

## 3. Major Polymers and Biosynthesis of Cell Walls

**Figure 2:**
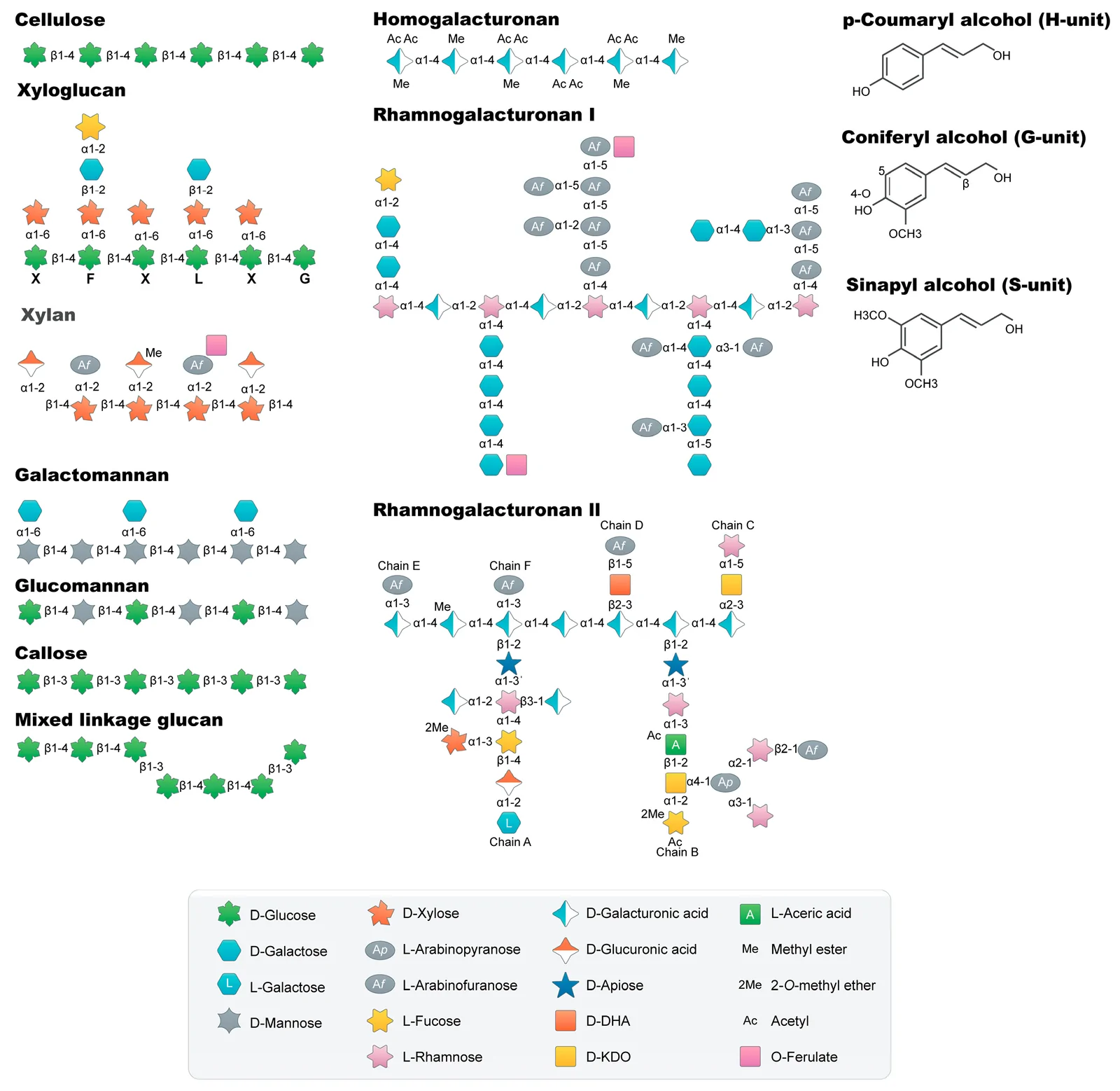
Representative structures of major plant cell wall polysaccharides and monolignols. Representative structures include cellulose, hemicelluloses (xyloglucan, xylan, galactomannan, glucomannan, and mixed-linkage glucan), pectin (homogalacturonan, rhamnogalacturonan I, and rhamnogalacturonan II), callose, and monolignols (p-coumaryl alcohol, coniferyl alcohol, and sinapyl alcohol). Monosaccharide symbols are defined at the bottom.

Plant cell walls are complex structural matrices composed primarily of polysaccharides, lignin, proteins, and diverse minor components (Figure 2). Among these constituents, polysaccharides and lignin form the major structural framework, whereas wall-associated proteins and enzymes contribute to wall assembly, modification, and signaling. Plant cell wall assembly requires the coordinated biosynthesis, trafficking, deposition, and remodeling of multiple polymer classes. Cellulose is synthesized at the plasma membrane by cellulose synthase complexes, where newly formed β-1,4-glucan chains crystallize into microfibrils as they are extruded into the wall. These microfibrils provide the main load-bearing framework. In contrast, most matrix polysaccharides, including hemicelluloses and pectins, are synthesized in the Golgi apparatus and delivered to the cell surface through vesicle trafficking. After secretion, they interact with cellulose and with one another to regulate wall hydration, porosity, extensibility, adhesion, and overall architecture. In secondary walls, lignin precursors are also exported to the wall and polymerized extracellularly, adding rigidity, hydrophobicity, and resistance to degradation. Thus, wall assembly is an integrated process involving polymer synthesis, intracellular transport, cytoskeletal guidance, extracellular polymer interactions, and remodeling by wall-associated enzymes and proteins.

### 3.1. Cellulose: The Principal Load-Bearing Framework

Cellulose is the principal load-bearing polysaccharide of plant cell walls and a major determinant of wall strength, anisotropy, and growth. It is synthesized at the plasma membrane by cellulose synthase (CesA) rosette complexes, whose trajectories are guided by cortical microtubules, thereby linking cytoskeletal organization to the orientation of newly deposited microfibrils [18,19,20]. In primary walls, cellulose microfibrils are embedded in a hydrated, extensible matrix that permits growth, whereas in secondary walls they are deposited in thicker, highly ordered layers that provide mechanical support and enable xylem function under transpirational tension. Distinct CesA isoforms mediate cellulose synthesis in primary and secondary walls, making cellulose biosynthesis central to wall assembly and plant morphogenesis.

#### 3.1.1. Molecular Components of Cellulose Biosynthesis

Cellulose consists of linear β-1,4-linked glucan chains that assemble into semi-crystalline microfibrils, providing tensile strength and mechanical anisotropy to plant cell walls. Cellulose is synthesized at the plasma membrane by cellulose synthase A proteins, CesAs, which are membrane-integrated glycosyltransferases organized into large cellulose synthase complexes, CSCs. The composition of CSCs varies according to wall type, cell identity, and developmental context, allowing cellulose production to be adapted to cell expansion in primary walls or mechanical reinforcement in secondary walls.

Genetic evidence for CesA function was first established through the Arabidopsis temperature-sensitive radial swelling 1, *rsw1*, mutant, which is defective in primary wall cellulose synthesis and carries a mutation in *AtCESA1* [18]. Genome-wide analyses later identified ten CESA genes in Arabidopsis, together with multiple cellulose-synthase-like (CSL) gene families, some of which encode enzymes involved in the synthesis of hemicellulosic backbones rather than cellulose itself [21]. In Arabidopsis, primary wall cellulose synthesis generally requires CESA1, CESA3, and one of the partially redundant CESA2, CESA5, CESA6, or CESA9 proteins, whereas secondary wall cellulose synthesis depends mainly on CESA4, CESA7, and CESA8 [18,19]. This division between primary- and secondary-wall CesA isoforms highlights the developmental specialization of cellulose biosynthesis.

CSC activity is further regulated by auxiliary proteins, post-translational modifications, and trafficking factors. KORRIGAN, a membrane-associated endo-β-1,4-glucanase, has been implicated in cellulose synthesis and may help process abnormal or disordered glucan chains, although its exact biochemical role remains unresolved [22,23,24]. COBRA, a glycosylphosphatidylinositol-anchored protein, influences cellulose deposition, crystallinity, and oriented cell expansion [25,26]. In addition, STELLO proteins have been linked to Golgi-associated CSC assembly and secretion, whereas CSI1, PATROL1 and exocyst components contribute to efficient CSC delivery to the plasma membrane, indicating that cellulose synthesis is coordinated by a broader regulatory network beyond the CesA catalytic subunits themselves [27,28].

#### 3.1.2. Structural Organization of Cellulose Synthase Complexes

The discovery of hexagonal “rosette” structures in the plasma membrane by freeze-etching electron microscopy provided the first structural framework for understanding plant cellulose synthesis [29]. These rosettes were interpreted as cellulose synthase complexes capable of synthesizing multiple glucan chains simultaneously, thereby linking enzyme organization to cellulose microfibril formation. Mechanistic insight initially came from bacterial cellulose synthase systems: crystal structures of the bacterial BcsA-BcsB complex revealed the conserved principles of processive β-1,4-glucan synthesis, including a glycosyltransferase catalytic domain, a transmembrane glucan-conducting channel, and regulatory elements controlling polymer translocation [30,31]. High-resolution structural studies have substantially refined models of plant CSC organization. Cryo-EM analysis of recombinant PttCESA8 revealed a plant CesA homotrimer in which each subunit contains seven transmembrane helices and an independent glucan-conducting channel, with the three glucan chains converging toward a common extracellular exit region [32]. This structure supports the idea that CesA trimers represent fundamental building blocks of the larger rosette CSC. However, because the resolved structure represents a recombinant homotrimer rather than an intact native heteromeric rosette, the precise arrangement of distinct CesA isoforms within functional CSCs remains unresolved. Current models propose either that each rosette lobe contains an isoform-specific homotrimer or that each lobe is composed of a heterotrimer containing the three CesA isoforms required for a given wall type, indicating that the organization of functional CSCs may vary with isoform composition and developmental context.

CSC architecture is directly related to cellulose microfibril formation, but the exact relationship between rosette organization and microfibril chain number remains debated. An approximately 18-chain microfibril model is widely accepted for many primary walls, whereas alternative models, including 24-chain microfibrils, have been proposed for certain secondary walls such as wood [33,34]. These differences suggest that microfibril architecture may vary among species, tissues, developmental stages, and wall types. Resolving how CSC organization determines glucan chain number, crystallinity, and lateral aggregation will require integration of native CSC structural studies, genetic analysis of isoform-specific assembly, and high-resolution biophysical characterization of cellulose microfibrils.

#### 3.1.3. Cytoskeletal Guidance, Trafficking, and Cellulose Microfibril Orientation

Cellulose synthase complexes are assembled within the endomembrane system and delivered to the plasma membrane through Golgi/TGN-dependent secretory trafficking. After insertion, CSCs move within the plasma membrane as they synthesize glucan chains; this movement is driven primarily by cellulose polymerization rather than by microtubule-based motor activity. The connection between CSCs and cortical microtubules is mediated in large part by CELLULOSE SYNTHASE INTERACTIVE1/POM2, CSI1/POM2, which interacts with both CesA complexes and microtubules and is required for efficient microtubule-guided CSC motility [35]. Under stress conditions, COMPANION OF CELLULOSE SYNTHASE, CC, proteins help maintain CSC-microtubule association and support cellulose synthesis when cortical microtubules are destabilized, for example during salt stress [36]. Additional trafficking factors, including STELLO, PATROL1, and exocyst components, further regulate CSC assembly, secretion, and plasma membrane delivery, emphasizing that cellulose deposition depends on coordinated control of CSC biogenesis, insertion, motility, and turnover [27,28].

### 3.2. Hemicellulose Biosynthesis in the Golgi

#### 3.2.1. Structural Diversity and Assembly of Hemicelluloses

Hemicelluloses comprise a diverse group of matrix polysaccharides, including xyloglucans, xylans, mannans, and mixed-linkage glucans (MLGs) [1]. Although a strict definition of hemicellulose remains difficult, these polymers are generally characterized by their ability to associate with cellulose microfibrils and thereby influence wall architecture, mechanics, and porosity. Most hemicelluloses contain β-1,4-linked backbones that are decorated with glycosyl, acetyl, or phenolic substituents, whereas MLGs are unusual in containing both β-1,4 and β-1,3 linkages and lacking side-chain substitutions. The diversity of backbone composition and substitution pattern determines the physicochemical properties, cellulose-binding behavior, and biological functions of different hemicelluloses [37].

Hemicellulose composition also varies markedly across plant lineages and wall types. Xyloglucan (XyG) is typically abundant in Type I primary walls of dicots, gymnosperms, and non-commelinid monocots, whereas xylans and MLGs are especially prominent in grasses, and xylans dominate many secondary walls. Mannans, including glucomannans and galactomannans, are particularly enriched in gymnosperm secondary walls and seed endosperm. This distribution reflects both evolutionary diversification and specialized wall functions.

Most hemicellulosic polysaccharides are synthesized in the Golgi apparatus by coordinated activities of membrane-associated glycosyltransferases, cellulose synthase-like proteins, acetyltransferases, and nucleotide–sugar transport systems. Backbones of xyloglucans, mannans, and MLGs are mainly synthesized by cellulose synthase-like (CSL) proteins belonging to the GT2 family, whereas xylan backbones are synthesized by type II membrane proteins from the GT43 and GT47 families rather than by CSL proteins [21,37,38,39,40]. Thus, hemicellulose biosynthesis is best viewed as a Golgi-centered process integrating backbone synthesis, side-chain addition, acetylation, and intracellular trafficking (Table 1).

#### 3.2.2. Biosynthesis of Specific Hemicellulose Classes

##### Xyloglucans

Xyloglucans, XyGs, are structurally complex hemicelluloses with a β-1,4-linked glucan backbone similar to cellulose, but decorated with α-linked xylosyl residues at the O-6 position of glucosyl units. Many xylosyl residues are further substituted with galactosyl, fucosyl, arabinosyl, or galacturonosyl residues, generating species- and tissue-specific side-chain patterns commonly described using single-letter nomenclature [37]. XyG is the major hemicellulose in the primary walls of gymnosperms, dicots, and non-commelinid monocots, which are generally classified as Type I primary walls [37].

The XyG backbone is synthesized by cellulose synthase-like C (CslC) proteins. In Arabidopsis, *cslc4* and higher-order *cslc* mutants show strongly reduced or undetectable XyG levels, supporting the essential role of CslC proteins in XyG backbone synthesis [41,42]. Xylosylation is initiated by XXT1, XXT2, and XXT5, while additional side-chain elaboration involves enzymes such as MUR3, XLT2, XST1/XST2, XUT1, and FUT1, which add galactosyl, arabinosyl, galacturonosyl, or fucosyl residues in a position-specific manner [43,44,45,46,47,48]. Importantly, severe XyG reduction does not necessarily abolish viability: Arabidopsis mutants with strongly diminished XyG can partially compensate through remodeling of other wall components, highlighting the plasticity of primary wall networks [42,58].

##### Xylans

Xylans consist of a β-1,4-linked xylosyl backbone substituted with glucuronic acid, methylglucuronic acid, arabinose, acetyl groups, and, in grasses, ferulate or other phenolic residues. Xylans are the dominant hemicelluloses in angiosperm secondary walls and in grass primary walls, corresponding to Type II primary wall systems [37]. Because of their abundance in woody biomass and grass cell walls, xylans represent one of the largest reservoirs of fixed carbon in the biosphere and strongly influence secondary wall architecture, biomass recalcitrance, and industrial processing properties [2,3].

Unlike xyloglucans and mannans, xylan backbones are not synthesized by CSL proteins. Instead, genetic and biochemical studies have identified IRX10 and IRX10L, members of the GT47 family, as β-1,4-xylosyltransferases required for xylan backbone elongation, while GT43 proteins IRX9, IRX9L, IRX14, and IRX14L are also required for normal xylan synthesis and may function as part of a xylan synthase complex [38,39,40,49]. Dicot xylans contain a characteristic reducing-end oligosaccharide, whose biosynthesis involves genes such as *IRX7/FRA8*, *IRX8/GAUT12*, and *PARVUS/GATL1*, although the biological function of this structure remains incompletely understood [59,60]. In grasses, xylan arabinosyl residues can also be feruloylated, creating sites for oxidative coupling and cross-linking that are important for wall strength and biomass recalcitrance [2,3,37].

##### Mannans

Mannans, including galactomannans and glucomannans, are β-1,4-linked mannosyl-rich polysaccharides that are often substituted with galactosyl residues and may also be acetylated [51]. Although generally less abundant than xylans and other matrix polysaccharides in the primary and secondary walls of most angiosperms, they are a predominant hemicellulose class in gymnosperm secondary walls. Mannans are also highly enriched in seed endosperm, where galactomannans and glucomannans are major components [51]. CSLA family members are responsible for mannan and glucomannan backbone biosynthesis [50], and subsequent studies in other species further supported their conserved role as mannan synthases [51]. In addition to backbone formation, galactomannan galactosyl transferase (GMGT) appears to participate in side-chain elaboration [52], whereas mannan O-acetyltransferases (MOATs) catalyze O-acetylation of the mannan backbone by modifying mannosyl residues with acetyl groups [53].

##### Mixed-Linkage Glucans (MLGs)

MLGs are hemicelluloses characterized by a relatively simple structure: a glucan backbone containing both β-1,4 and β-1,3 linkages and typically lacking side-chain substitutions. The β-1,3 linkages introduce bends into the otherwise linear glucan chain, and the frequency and distribution of these linkages strongly influence polymer conformation, solubility, and wall properties [37]. MLGs are especially abundant in grass primary walls and are also found in some lower plants and fungi, but they are absent or present only at very low levels in most dicot primary walls.

MLG synthesis is mainly associated with CslF and CslH proteins. Expression of rice CslF6 in Arabidopsis, which lacks endogenous CslF proteins, is sufficient to induce β-1,3;1,4-glucan accumulation, demonstrating that CslF proteins can synthesize MLG [54,55]. CslH proteins represent an additional enzyme group capable of contributing to MLG synthesis in grasses [56]. Unlike many other hemicelluloses, MLG biosynthesis may not be restricted to the Golgi apparatus, as CslF6 has been reported to localize to both Golgi-associated compartments and the plasma membrane, suggesting that MLG synthesis may occur at multiple cellular sites [57]. This makes MLG an important exception within the broader framework of Golgi-centered hemicellulose biosynthesis and indicates that the cellular mechanisms controlling its synthesis remain unresolved.

### 3.3. Pectin Biosynthesis, Modification, and Wall Integration

Pectins are among the most structurally complex glycans in plant cell walls and are particularly enriched in primary walls and the middle lamella. Their functional diversity arises from variation in backbone architecture, side-chain composition, methylesterification, acetylation, calcium binding, and post-synthetic remodeling. The major pectic domains include homogalacturonan, HG, rhamnogalacturonan I, RG-I, and rhamnogalacturonan II, RG-II, together with less abundant substituted forms such as xylogalacturonan [61]. HG is composed of α-1,4-linked galacturonic acid residues whose carboxyl groups can be methylesterified, whereas RG-I contains a repeating rhamnose-galacturonic acid backbone decorated with arabinan, galactan, and arabinogalactan side chains. RG-II, although less abundant, is structurally intricate and forms borate diester crosslinks that contribute to wall integrity and normal plant growth [61]. Through these structural features, pectins regulate wall hydration, porosity, cell adhesion, mechanical properties, signaling, defense responses, fruit texture, organ abscission, and reproductive development [61].

Pectin biosynthesis occurs predominantly in the Golgi apparatus and requires the coordinated activities of glycosyltransferases, methyltransferases, acetyltransferases, and nucleotide–sugar transport systems [61]. HG biosynthesis is mediated by galacturonosyltransferases, including members of the GAUT family, which can synthesize polymeric HG in vitro [62,63]. RG-I biosynthesis requires enzymes responsible for backbone elongation and side-chain addition, and studies of Arabidopsis seed mucilage have identified key components involved in RG-I assembly, including rhamnosyltransferases required for backbone formation [64]. Compared with HG, the enzymatic machinery responsible for RG-I side-chain construction and RG-II assembly remains less completely resolved, reflecting the high structural complexity of pectic polysaccharides [61]. Similar to hemicellulose biosynthesis, pectin production depends on the availability of activated nucleotide–sugar donors and their transport into the Golgi lumen. During Golgi assembly, pectins also undergo methylesterification and acetylation, generating highly modified polymers that are subsequently secreted to the cell wall.

After secretion, pectins are extensively remodeled in the apoplast. The degree and spatial pattern of HG methylesterification are particularly important for wall mechanics. Highly methylesterified HG has a limited capacity to form calcium-mediated crosslinks, whereas blockwise de-methylesterification promotes Ca^2+^-dependent “egg-box” structures that stiffen and stabilize the wall [65,66]. In contrast, other de-methylesterification patterns can increase susceptibility to pectin-degrading enzymes and thereby facilitate wall loosening, tissue separation, or developmental remodeling. These modifications generate spatially patterned pectin domains with distinct mechanical and signaling properties. For example, localized pectin de-methylesterification can influence microtubule organization and indirectly affect cellulose microfibril orientation, illustrating how pectin remodeling is integrated with broader cell wall architecture [61].

### 3.4. Lignin

#### 3.4.1. Structure, Composition, and Chemical Characterization

Lignin is a major phenolic polymer of plant secondary cell walls, where it contributes mechanical strength, hydrophobicity, and resistance to biological degradation. It is formed primarily from three hydroxycinnamyl alcohols-*p*-coumaryl, coniferyl, and sinapyl alcohols-which give rise to hydroxyphenyl (H), guaiacyl (G), and syringyl (S) units, respectively. These monolignols are mainly derived from the phenylpropanoid pathway and are incorporated into the wall through oxidative radical coupling [67]. Lignin composition varies substantially among plant lineages and cell types. Gymnosperm lignins are dominated by G units, whereas angiosperm lignins typically contain both G and S units with relatively low levels of H units. Grass lignins are more compositionally diverse and often include hydroxycinnamate-derived decorations, such as *p*-coumarates and ferulates, as well as noncanonical units including tricin [67].

Unlike cellulose and most hemicelluloses, lignin lacks a defined primary sequence and is generated through combinatorial radical coupling. Its structure is therefore shaped by monolignol supply, oxidative chemistry, wall environment, and the spatial activity of polymerizing enzymes. Rather than forming a uniform hyper-crosslinked network, lignin is now regarded as a heterogeneous polymer whose chain length, branching pattern, and crosslink density vary with monomer composition and wall context, particularly the S/G ratio [67].

#### 3.4.2. Monolignol Biosynthesis, Pathway Flexibility, and Compositional Plasticity

The classical model of lignin biosynthesis described a phenylpropanoid metabolic grid in which trans-cinnamic acid is converted through multiple routes into the three major monolignols that generate H, G, and S lignin units [68]. Subsequent biochemical, genetic, and metabolic flux analyses refined this view by revealing both pathway specificity and substantial flexibility in monolignol production [69,70]. Most canonical monolignol biosynthetic genes have now been isolated and functionally validated, including those encoding PAL, C4H, 4CL, HCT, C3H, CSE, CCoAOMT, CCR, F5H, COMT, and CAD activities [71].

Lignin also exhibits remarkable compositional plasticity. In addition to the canonical H, G, and S units, natural lignins can incorporate noncanonical monomers such as tricin, stilbenes, and feruloyl putrescine, depending on species, tissue, and metabolic context [67]. This plasticity has enabled lignin engineering strategies aimed at improving biomass processing or generating value-added products. For example, monolignol ferulates and other engineered monomers can introduce more labile linkages into lignin and enhance saccharification efficiency [72]. Similarly, increasing *p*-hydroxybenzoate incorporation in poplar lignin has been proposed as a strategy to produce “clip-off” aromatic molecules for downstream valorization [73]. However, lignin engineering often involves trade-offs among wall integrity, plant growth, stress resistance, and processing performance, making precise spatial and developmental control a central challenge.

Lignin biosynthesis is also tightly coordinated with secondary wall formation through transcriptional regulatory networks. NAC and MYB transcription factors form hierarchical regulatory modules that control secondary wall biosynthesis, including cellulose, hemicellulose, and lignin pathway genes [8]. This coordination helps ensure that monolignol production, polysaccharide deposition, and wall thickening occur in appropriate cell types and developmental stages.

#### 3.4.3. Oxidative Polymerization and Spatial Control of Lignification

Lignin polymerization occurs in the apoplast and is driven by oxidative enzymes that generate monolignol radicals for coupling. Class III peroxidases were initially regarded as the principal catalysts because of their cell wall localization and their ability to produce lignin-like dehydrogenation polymers in vitro, whereas laccases were later identified as equally important contributors through genetic and biochemical studies [74,75,76]. Current evidence supports a model in which both peroxidases and laccases participate in lignification, with their relative contributions varying according to cell type, developmental stage, and wall domain. For example, peroxidase activity is essential for Casparian strip lignification, whereas laccases play prominent roles in secondary wall lignification in vascular tissues [77,78]. Importantly, lignification is not simply a bulk chemical reaction but a spatially organized process controlled by the localization of oxidative enzymes, the availability of hydrogen peroxide, and the physicochemical properties of the surrounding wall matrix. PEROXIDASE 64 preferentially accumulates at cell corners, where lignification often initiates, whereas LACCASE 4 is enriched in secondary wall domains, indicating that distinct oxidative machineries operate in different wall regions [79]. The Casparian strip provides a particularly clear example of how lignin deposition can be confined to a sharply defined wall domain through precise spatial regulation of polymerization [80]. Although lignin formation is chemically less templated than polysaccharide biosynthesis, plants nevertheless exert substantial control over when and where polymerization occurs through coordinated regulation of oxidative enzymes, reactive oxygen supply, and wall context. Lignification should therefore be viewed as a chemically permissive but tightly regulated assembly process embedded within secondary wall development. Beyond its spatial regulation, the nanoscale structuration of lignin, including molecular conformation, aggregation state, and supramolecular organization, substantially influences its physicochemical properties, particularly adhesion. Such structure-property relationships are not only biologically relevant for wall assembly but also critical for industrial applications such as bio-based adhesives and wood composites [81,82].

### 3.5. Coordination of Polymer Deposition and Structural Integration

**Figure 3:**
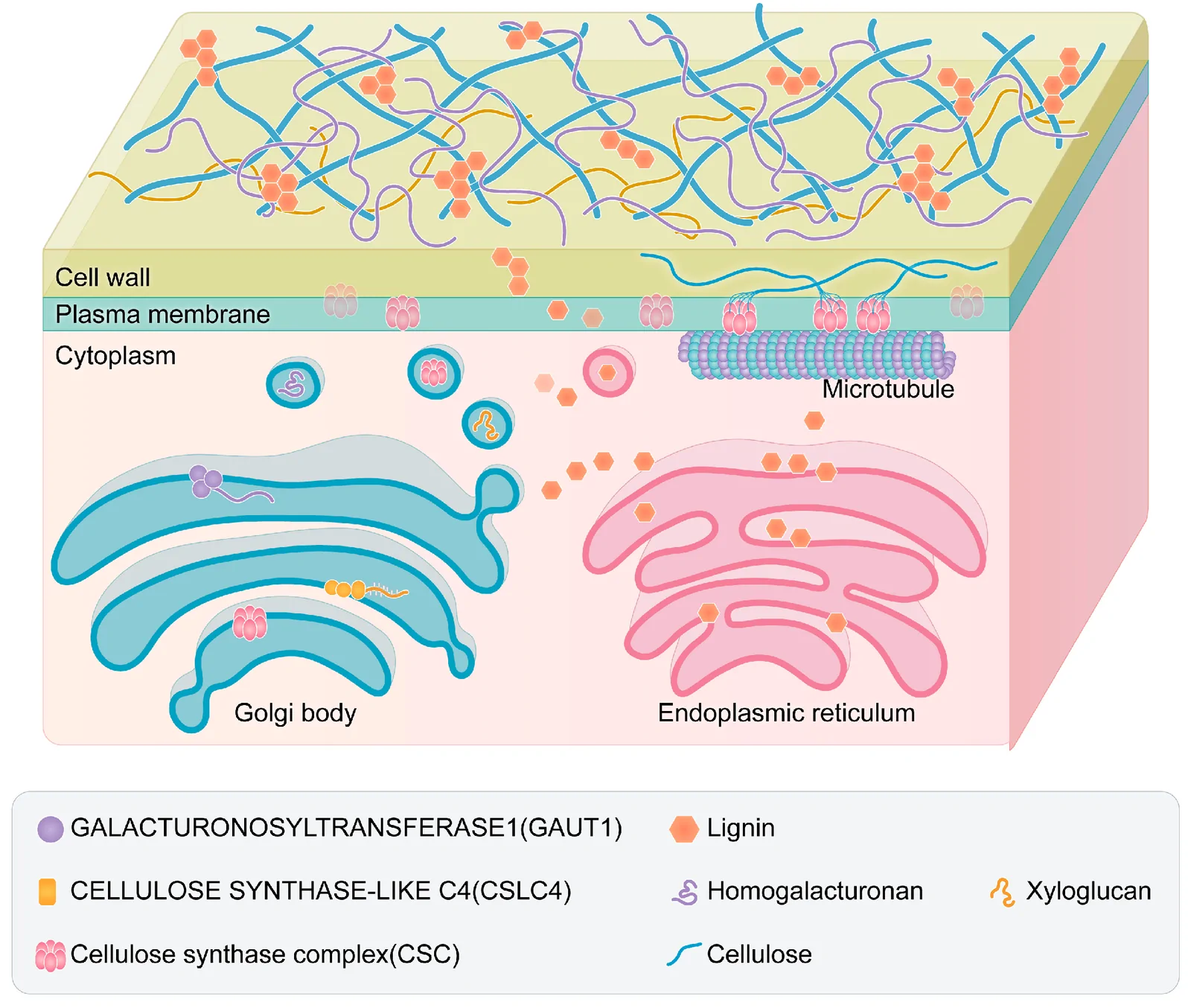
Plant cell wall structure and assembly: Cellulose synthase complexes (CSCs, pink) are delivered from the Golgi apparatus to the cortical plasma membrane, where cortical microtubules help coordinate their insertion. Once inserted, CSCs synthesize cellulose and move laterally within the membrane under the guidance of cortical microtubules, driven by cellulose polymerization. Matrix polysaccharides, including hemicelluloses (orange) and pectins (purple), are synthesized in the Golgi apparatus by glycosyltransferases and may be further modified by the addition of side chains. Monolignols are synthesized in the cytosol and subsequently exported to the apoplast, where they are oxidatively polymerized to form lignin. In the apoplast, cellulose, matrix polysaccharides, and lignin interact to form a structurally integrated and dynamic cell wall network that provides mechanical support, regulates wall extensibility and porosity, and contributes to growth control and stress adaptation.

Although the biosynthesis of individual wall polymers has been increasingly well characterized, it remains more difficult to understand how these polymers are deposited, arranged, and integrated into a functional cell wall (Figure 3). Early models emphasized covalent or physical cross-linking among matrix polysaccharides based largely on enzymatic degradation products [83]. Subsequent biochemical and ultrastructural studies led to the distinction between type I walls, typical of dicot primary walls, and type II walls, characteristic of grasses and other commelinid monocots [6]. More recently, solid-state NMR, atomic force microscopy, and genetic analyses have substantially revised these classical views. In particular, the long-standing model in which xyloglucan serves as the dominant load-bearing tether between cellulose microfibrils in type I primary walls has been challenged by evidence from Arabidopsis xyloglucan-deficient mutants, which show only moderate developmental defects [6,42]. Current models instead emphasize cellulose–pectin interactions as key contributors to primary wall mechanics, whereas cellulose–xylan interactions provide a major structural framework in secondary walls. In grass primary walls, where glucuronoarabinoxylan is the predominant hemicellulose, xylan-related interactions also play major architectural roles [84,85].

A central challenge in wall assembly is that different polymers are synthesized in different cellular compartments and must be coordinated during deposition at the cell surface. Cellulose is produced at the plasma membrane by cellulose synthase complexes, where nascent glucan chains assemble into microfibrils, whereas most hemicelluloses and pectins are synthesized in the Golgi and delivered by secretory trafficking. Proper cellulose organization depends not only on CESA activity and cellulose synthase complex (CSC) behavior but also on auxiliary proteins such as the COBRA-like protein BC1, as indicated by mutants with altered microfibril crystallinity, morphology, or crystallite size [32,86]. Cortical microtubules guide CSC trajectories and thereby influence cellulose orientation, while vesicle trafficking likely helps position matrix polysaccharides relative to sites of cellulose deposition [87]. After deposition, matrix polysaccharides and lignin further shape cellulose organization and convert polymer patterns into mechanically functional wall architectures. Perturbation of arabinoxylan, pectins, or xyloglucan can alter cellulose ordering, spacing, and mechanics, indicating that these polymers actively regulate the cellulose framework rather than merely filling the wall matrix [58,85,88]. In secondary walls, xylan can bind directly to crystalline cellulose in a two-fold screw conformation, forming an ordered cellulose–xylan scaffold that contributes to multiscale organization and mechanical stability [84,85,89]. Lignin is subsequently integrated into this scaffold, increasing rigidity, hydrophobicity, and resistance to enzymatic deconstruction [80]. AFM observations of stress-induced kinking, shearing, separation, and sliding of cellulose microfibrils further support the idea that nanoscale rearrangements contribute to wall extensibility and remodeling [90]. Together, these findings show that cell wall structure emerges from coordinated polymer synthesis, secretion, cytoskeletal guidance, polymer interactions, lignification, and mechanical feedback, although how these processes are coupled across cell types, developmental stages, and environmental conditions remains incompletely understood.

## 4. Cell Wall Integrity and Signal Transduction

The plant cell wall is now understood not merely as a structural matrix but as a dynamic signaling compartment that continuously reports the physical and chemical status of the cell surface. Because plant cells are encased in walls and cannot move away from unfavorable conditions, they must rapidly detect and respond to changes in wall mechanics, polymer organization, and extracellular stress. Cell wall integrity (CWI) therefore refers to the capacity of cells to monitor whether the wall remains mechanically and chemically functional and to activate compensatory responses when integrity is compromised. CWI signaling operates during normal growth, when wall loosening must be balanced with turgor-driven expansion, and becomes particularly important under drought, salinity, wounding, pathogen invasion, and mechanical strain. Although most studies have focused on primary walls, secondary wall perturbation can also trigger CWI-related responses, which is especially relevant in lignin-engineered plants. Reduced lignin may improve biomass digestibility but can simultaneously activate defense-like responses and compromise growth; in some lignin-modified lines, pathogenesis-related proteins and other defense markers are ectopically induced [91]. Importantly, growth–defense relationships are context-dependent, as some suppressor mutants restore growth in low-lignin backgrounds while retaining defense gene expression [92], suggesting that these outputs can be partially uncoupled.

### 4.1. Causes and Consequences of Cell Wall Integrity Loss

CWI loss can arise from defects in cellulose synthesis, pectin remodeling, hemicellulose organization, lignification, wall protein function, or mechanical coupling among the wall, plasma membrane, and cytoskeleton. Among these, disruption of cellulose synthesis has provided one of the most informative experimental systems for studying CWI responses, because cellulose is a major load-bearing component of primary walls and its reduction directly alters the mechanical balance between turgor pressure and wall resistance. Early studies showed that plant cells could gradually adapt to the cellulose synthesis inhibitor DCB through compensatory cell wall remodeling: some cells produced pectin- and protein-rich walls, whereas others increased phenolic cross-linking of arabinoxylans [93,94]. These findings provided early evidence that cells actively perceive wall architectural defects and initiate compensatory responses rather than passively tolerate polymer loss. Consistent with this view, Arabidopsis treated with isoxaben, which disrupts cellulose synthase complex function, has become a canonical model for CWI signaling. Isoxaben reduces cellulose deposition, alters cell expansion, promotes pectin accumulation, induces callose and sometimes ectopic lignification, and modulates wall-related gene expression, including genes encoding CSLD5 and glycine-rich proteins [95]. Genetic studies further identified THESEUS1 as a receptor-like kinase involved in responses to cellulose deficiency, providing a direct link between cellulose perturbation and plasma membrane signaling [96]. Beyond changes in cellulose content, natural stresses such as tensile stress, osmotic stress, and salinity can perturb CWI through distinct mechanical and biochemical mechanisms. Thus, CWI loss is best understood as a combined mechanical and biochemical disturbance rather than as a defect in any single polymer.

### 4.2. Wall-Derived Oligosaccharides and Peptide Signals at the Cell Wall-Plasma Membrane Interface

The wall communicates its structural status in part through the release of wall-derived oligosaccharides, which act as damage-associated or remodeling-associated molecular signals. During development or stress, wall-modifying enzymes, pathogen-secreted hydrolases, or physical damage generate fragments from pectins, cellulose, hemicelluloses, or mixed-linkage glucans. Pectin-derived oligogalacturonides are among the best-characterized wall-derived signals and are associated with defense activation, calcium signaling, reactive oxygen species production, and transcriptional reprogramming [97]. Cellulose-derived cellodextrins can also act as damage-associated molecular patterns, linking cellulose breakdown to immune and stress signaling [98]. Other oligosaccharides, including fragments from xyloglucans, xylans, or grass mixed-linkage glucans, may also convey information about wall architecture, although their biological specificity, cognate receptors, and physiological concentration ranges remain less clearly defined [99]. A central unresolved question is how plants distinguish normal wall turnover during growth from damage-associated fragmentation during stress, a distinction that likely depends on the identity, abundance, modification state, and temporal dynamics of specific oligosaccharide species.

Peptide signals provide a complementary layer of communication at the wall-plasma membrane interface. Some peptides are secreted into the apoplast and function near the wall, where they regulate growth, mechanosensing, immunity, and stress responses. RALF peptides are closely associated with FERONIA-related pathways and can influence wall pH, cell expansion, receptor activity, and stress signaling [100]. Plant elicitor peptides amplify immune responses after tissue damage or pathogen attack through PEPR-mediated perception, whereas other peptide families contribute to development, tissue patterning, or local cell wall remodeling [101]. These peptides do not act in isolation: their mobility, stability, receptor accessibility, and local concentration are shaped by wall porosity, pH, charge, and polymer composition. Wall-derived oligosaccharides and apoplastic peptides therefore form complementary signal classes. The former often report polymer damage or remodeling, whereas the latter tune receptor-mediated responses at the cell surface and help coordinate local repair with broader developmental or immune programs.

### 4.3. Plasma Membrane Receptors

CWI signaling depends fundamentally on plasma membrane-localized receptors that translate extracellular wall status into intracellular responses. Several receptor-like kinases and related proteins have been implicated, including FERONIA, THESEUS1, wall-associated kinases (WAKs), PEPRs, and other RLKs (Table 2). These receptors may detect wall-derived ligands, peptide signals, altered polymer status, or mechanical changes transmitted across the wall-plasma membrane continuum. FERONIA and related CrRLK1L-family proteins are particularly important because they participate in growth regulation, mechanotransduction, reproduction, immunity, and stress responses. FERONIA can perceive RALF peptides and modulate apoplastic pH, cell expansion, ROS production, and hormone-related signaling [100]. THESEUS1 is strongly associated with responses to cellulose deficiency and has been proposed to function as part of a surveillance mechanism that links impaired wall biosynthesis to altered growth and defense outputs [96]. WAKs provide a plausible connection between pectin status and intracellular signaling because they can bind pectin-derived fragments and participate in defense responses [5,102,103]. PEPRs contribute to damage and defense amplification through perception of endogenous plant elicitor peptides [101]. This distributed receptor architecture suggests that CWI perception is not controlled by a single universal sensor but emerges from the coordinated activity of multiple receptor modules with partly overlapping inputs and outputs.

### 4.4. Integration of Growth, Stress, Defense, and Hormonal Signaling

CWI signaling is deeply integrated with hormone networks that coordinate growth, stress adaptation, and immunity. Auxin, brassinosteroids, gibberellins, ethylene, jasmonates, salicylic acid, and abscisic acid can all influence wall metabolism or CWI outputs, but their effects are highly context-dependent [124,125,126,127,128,129,130]. Growth-promoting hormones generally favor wall loosening, expansion, and biosynthesis, whereas stress- and defense-associated hormones often restrict growth and promote reinforcement, although this distinction is not absolute. For example, cell wall perturbation can activate jasmonate, ethylene, or salicylic acid pathways, while drought and salinity may engage ABA-dependent responses that modify wall stiffness, pectin chemistry, or cell expansion [126]. Conversely, hormonal status can alter the sensitivity of cells to wall defects by changing wall composition, receptor abundance, cytoskeletal organization, or the balance between expansion and reinforcement.

A defining feature of CWI signaling is that it operates as a network of partially overlapping modules rather than as a linear pathway. Diverse stress-associated cell wall perturbation, including cellulose deficiency, pectin degradation, and altered secondary wall composition can activate partially distinct sensing pathways at the cell surface. These pathways may involve receptors or signaling modules such as FERONIA, THESEUS1, WAKs, and PEPRs, depending on the nature of the stimulus [5]. Although the initiating cues differ, their downstream effects often converge on common signaling outputs, including Ca^2+^ dynamics, ROS production, apoplastic pH changes, and cytoskeletal reorganization, which are further integrated through hormone crosstalk to coordinate growth, wall repair, and defense. This network architecture allows plants to adjust wall construction and defense according to developmental stage, cell type, and environmental context. Current evidence indicates that CWI signaling is highly context-dependent, requiring cells to distinguish growth-associated wall remodeling from damage-related perturbation, to integrate mechanical cues with wall-derived chemical signals, and to coordinate local wall responses with broader tissue-level outputs. In addition, activation of these pathways is often accompanied by growth–defense trade-offs, particularly in plants with altered lignin deposition or modified wall composition. Together, these features underscore the role of the plant cell wall as a dynamic signaling interface that links structural status to growth, repair, and defense responses.

## 5. Cell Wall Functions in Plant Physiology

The plant cell wall is a multifunctional structural matrix that supports plant growth, development, transport, and environmental adaptation. In addition to providing mechanical strength, the wall functions as a dynamic physiological interface that regulates cell expansion, tissue morphogenesis, water and solute movement, ion homeostasis, and defense. Because plants are sessile organisms, their walls must continuously adjust to changing internal and external conditions, including growth-generated mechanical stress, drought, salinity, nutrient imbalance, temperature fluctuation, heavy metal toxicity, pathogen invasion, and wounding. These adjustments depend on coordinated changes in wall composition, architecture, stiffness, porosity, and signaling capacity. Thus, the cell wall should be viewed not only as a structural scaffold but also as an active physiological system that links extracellular constraints with cellular and whole-plant responses [5].

The physiological roles of the wall can be organized around several interconnected functions. First, secondary cell walls provide mechanical support for upright growth, vascular stability, and xylem-mediated long-distance transport. Second, primary walls control cell expansion, organ morphogenesis, and tissue repair through biomechanical regulation and polymer remodeling. Third, wall remodeling contributes to abiotic stress adaptation by modifying hydration, extensibility, porosity, ion binding, and apoplastic transport. Fourth, specialized wall-derived barriers, such as the Casparian strip and suberized cell walls, regulate water and ion movement at the tissue level. Finally, the wall acts as a defensive interface against pathogens by providing a physical barrier, generating damage-associated signals, and supporting reinforcement responses. These functions are highly integrated: for example, salt stress can alter pectin cross-linking and wall porosity, xylem wall architecture affects both hydraulic conductance and yield, and pathogen-induced wall degradation can simultaneously trigger immune signaling and compensatory wall reinforcement.

### 5.1. Mechanical Support, Xylem Hydraulics, and Transport Efficiency

Upright growth, a defining adaptation of land plants, depends strongly on mechanically reinforced secondary cell walls in fibers, sclerenchyma, and xylem vessels. These walls are enriched in cellulose, hemicelluloses, lignin, and wall proteins, and their coordinated deposition and organization confer tensile strength, compression resistance, and tissue rigidity. In crops, compromised secondary wall formation often results in lodging, brittle culms, collapsed vessels, impaired transport, and reduced biomass or grain yield. Mutant analyses have demonstrated the importance of secondary wall integrity: fragile fiber mutants show defective interfascicular fibers with reduced tensile strength [131], whereas irregular xylem mutants exhibit collapsed vessels due to impaired cellulose, xylan, or lignin biosynthesis [19,38]. Rice brittle culm and brittle leaf sheath mutants further reveal the contribution of cellulose and xylan biosynthetic genes to culm strength and lodging resistance [132]. Altered xylan acetylation also disrupts secondary wall deposition in epidermal fibers and vessel cells, emphasizing that wall strength depends not only on polymer abundance but also on polymer modification and assembly [133]. At the nanoscale, reduced mechanical strength is associated with altered cellulose microfibril orientation, aberrant bundling, and disrupted cellulose–matrix interactions, as revealed by atomic force microscopy and other imaging approaches [85]. Thus, mechanical strength emerges from coordinated secondary wall deposition, lignification, microfibril organization, matrix polymer structure, and tissue-level patterning.

The same structural principles are essential for xylem hydraulics. Xylem vessels transport water and dissolved minerals under transpiration-driven negative pressure and therefore require walls that are both mechanically resistant and hydraulically efficient. Vessel secondary walls, composed of cellulose microfibrils embedded in hemicellulose and lignin matrices, prevent collapse under tension and provide waterproofing that supports long-distance water transport [8]. Defects in cellulose, xylan, or lignin synthesis frequently cause vessel collapse or reduced hydraulic function, demonstrating that polymer integrity is directly linked to water transport capacity [19,38,134]. Xylan structure and its interaction with cellulose further influence nanoscale wall organization and vessel stability [135,136].

Xylem transport is not determined solely by wall thickness or chemical composition; it also depends on patterned secondary wall deposition and pit architecture. Vessel walls contain spiral, reticulate, scalariform, or pitted secondary wall patterns that balance hydraulic conductivity with resistance to embolism spread. These patterns are established through cortical microtubule organization, local Rho-of-plants GTPase signaling, and targeted vesicle trafficking. For example, the ROP11-MIDD1-KIN13A module locally removes cortical microtubules to define pit regions, whereas microtubule-associated regulators and cytoskeletal patterning factors organize the spatial deposition of secondary walls [137,138,139]. A recent study further highlights the physiological significance of this process by showing that proper shaping of vessel wall pits is essential for maintaining xylem hydraulic performance and supporting grain yield [140]. This study identifies a xylan deacetylase that modulates pit geometry by reducing xylan acetylation, which in turn alters xylan–cellulose interactions at the nanoscale. These findings reveal how subtle chemical modifications of wall polymers can translate into whole-plant physiological outcomes. This finding is important because it links nanoscale or microscale wall patterning directly to whole-plant transport efficiency and agricultural productivity. In this view, vessel pits are not passive gaps in secondary walls but functional architectural elements that regulate the safety–efficiency balance of xylem transport.

### 5.2. Growth, Morphogenesis, Biomechanics, and Tissue Repair

Primary cell walls control plant growth by regulating the balance between turgor pressure and wall extensibility. Cell expansion requires localized wall loosening, polymer rearrangement, and controlled mechanical yielding, while excessive loosening must be avoided to maintain cell integrity. Expansins, xyloglucan endotransglucosylase/hydrolases, polygalacturonases, pectate lyases, pectin methylesterases, and other wall-modifying enzymes participate in this process by altering polymer interactions, pectin status, and wall mechanics [6,141]. Cellulose microfibril orientation is a major determinant of anisotropic growth: transverse microfibrils restrict radial expansion and promote longitudinal elongation, whereas changes in microfibril orientation can redirect growth patterns and cell shape [133]. Because cellulose synthase trajectories are coordinated with cortical microtubules, cytoskeletal organization provides a direct mechanism for translating intracellular polarity into extracellular wall architecture and directional growth [139].

Wall mechanics also contribute to organ morphogenesis. In meristems and developing organs, spatial variation in pectin methylesterification, calcium cross-linking, and wall stiffness helps define growth anisotropy and tissue curvature. The degree of pectin methylesterification in meristematic tissues regulates organ shape by modulating wall stiffness and growth direction. Similarly, the balance between esterified and de-esterified pectins influences lateral root initiation through the root clock mechanism [142]. Tissue-scale mechanics are also important: the epidermis often bears substantial tensile stress and can constrain or direct the growth of inner tissues, thereby acting as a mechanical coordinator of organ expansion [143]. Recent work has further revealed that spatially patterned pectin modification directly regulates stem cell dynamics through mechanical control of division plane orientation [144]. In the shoot apical meristem, pectin exhibits a bimodal methylesterification pattern: newly formed cross walls contain predominantly demethylesterified pectin and display lower stiffness, whereas mature walls are enriched in highly methylesterified pectin and exhibit greater stiffness [144]. This mechanical heterogeneity establishes a spatial landscape that guides stem-cell division orientation and maintains meristem homeostasis. The underlying pattern involves a nuclear sequestration mechanism in which *PME5* mRNA is retained in the nucleus by RZ-1B/1C proteins and released only upon nuclear envelope breakdown, ensuring spatiotemporally controlled pectin modification at newly forming walls [144]. Perturbation of this process causes stem-cell depletion and meristem termination, highlighting the importance of wall-level patterning in morphogenesis. This bimodal pattern is conserved in maize, soybean, and tomato, indicating broad relevance for crop architecture [144]. These observations show that plant morphogenesis is not determined only by genetic patterning or hormone gradients but also by the mechanical properties of the wall and their spatial regulation.

Tip-growing cells provide a clear example of dynamic wall remodeling during polarized growth. In pollen tubes and root hairs, highly methylesterified pectins are secreted at the growing apex to maintain wall flexibility, whereas subsequent pectin de-esterification, calcium cross-linking, cellulose deposition, and callose accumulation strengthen the subapical region [145]. Disruption of pectin methylesterase activity alters wall mechanical properties and inhibits pollen tube elongation, indicating that precise temporal control of wall stiffening and loosening is essential for sustained tip growth [145,146]. Recent models have further refined the classical turgor-driven view of expansion. The “expanding beam” model proposes that pectic nanofilaments may possess intrinsic expansion capacity, suggesting that cell walls can participate actively in growth rather than merely yielding passively to turgor pressure [147].

### 5.3. Cell Wall Remodeling in Abiotic Stress Adaptation

Abiotic stresses frequently disturb wall mechanics, hydration, porosity, ion balance, and apoplastic chemistry. In response, plants remodel wall composition and architecture to maintain growth, protect cellular integrity, and regulate transport. Under drought and osmotic stress, walls may become either stiffer or more extensible depending on tissue type, developmental stage, and stress intensity [148,149,150]. Wall stiffening can restrict water loss and protect cells from collapse, whereas controlled loosening can sustain root growth under reduced water availability. Changes in expansins, XTHs, pectin methylesterases, lignin deposition, suberin formation, and arabinogalactan proteins have all been associated with drought or osmotic stress responses [151]. Root walls are particularly important because root growth under water limitation depends on maintaining cell expansion while simultaneously adjusting apoplastic water flow and mechanical resistance.

Mechanical stress imposed by soil compaction also triggers specific cell wall remodeling responses [130]. Ethylene accumulates in compacted soil and activates the transcription factor OsARF1, which differentially regulates cellulose synthesis across root cell layers [130]. This differential regulation generates a “thicker epidermis-thinner cortex” biomechanical configuration: epidermal walls are reinforced for protection, whereas cortical walls are loosened to facilitate radial expansion. Together, these changes generate the axial growth force required for roots to penetrate compacted soil [130]. This study establishes a direct link between ethylene signaling, cell wall mechanics, and root adaptation to mechanical stress.

Salt stress illustrates the close connection between wall remodeling and ion homeostasis. High salinity alters pectin methylesterification, calcium cross-linking, cellulose synthesis, and cortical microtubule organization [36,106,152,153,154]. Because de-esterified pectins contain negatively charged carboxyl groups, they can bind cations such as Ca^2+^, Na^+^, and heavy metals, thereby influencing ion availability and apoplastic buffering [151]. Increased pectin demethylesterification and Ca^2+^ cross-linking may stiffen the wall and reduce excessive expansion, whereas altered pectin charge can affect Na^+^ retention in the apoplast [151,155]. However, these effects are context-dependent: excessive wall stiffening can inhibit growth, and uncontrolled pectin de-esterification can increase wall susceptibility to degradation. Thus, salt-induced wall remodeling reflects a balance between maintaining growth, protecting wall integrity, and regulating ion movement.

Temperature extremes also involve wall-based adaptation. Cold and freezing stress can alter wall hydration, and pectin status, affecting extracellular ice formation and cell survival. Changes in pectin methylesterification, xyloglucan metabolism, xylan modification, and lignification have been associated with cold acclimation and freezing tolerance [156,157,158,159]. Heat stress can disrupt wall loosening, cell expansion, and reproductive development, partly through altered wall enzyme activity and polymer dynamics [148,160,161]. Although the molecular details vary among species and tissues, these examples indicate that wall architecture contributes to thermal resilience by modulating mechanical stability, extracellular water behavior, and growth plasticity.

### 5.4. Cell Wall Functions in Pathogen Defense

The plant cell wall represents a dynamic defensive interface that both restricts microbial invasion and participates in immune signaling. The cell wall can limit pathogen penetration, while wall-associated antimicrobial compounds and specialized polymers further enhance defense [99]. To breach this barrier, pathogens often secrete cell wall-degrading enzymes, such as polygalacturonases, cellulases, xylanases, and pectate lyases. In response, plants reinforce the wall through callose deposition, lignification, phenolic cross-linking, extensin accumulation, and modifications of pectin structure. These structural reinforcements restrict pathogen spread and help compartmentalize damaged tissues. At the same time, wall degradation generates oligosaccharide fragments that function as damage-associated molecular patterns (DAMPs). Pectin-derived oligogalacturonides, cellulose-derived cellodextrins, and other wall fragments can activate immune responses, including calcium influx, reactive oxygen species production, MAPK activation, defense gene expression, and hormone-mediated responses [162]. Together, these processes underscore the role of the cell wall as a physiological surveillance interface, converting extracellular matrix damage into immune activation and enabling plants to detect pathogen attack at an early stage.

Wall-mediated defense is also closely linked to growth regulation. Reinforcement of the wall can improve resistance but may reduce extensibility and growth, whereas excessive wall loosening can support growth but increase susceptibility. Lignin engineering provides a clear example of this trade-off. Reduced lignin content may enhance biomass processing or digestibility but can also induce defense-like responses or impair mechanical stability. Conversely, compensatory pathways can sometimes restore growth while maintaining defense activation, indicating that growth–defense relationships are flexible rather than strictly antagonistic [92,163]. Thus, the wall serves as a central interface where mechanical support, immune defense, and resource allocation are balanced.

## 6. Knowledge Gaps and Future Perspectives

A central unresolved question in plant cell wall biology is how mechanical information is perceived and converted into biochemical responses. The wall is continuously exposed to turgor-driven expansion, tissue tension, wind, touch, and pathogen attack, yet the molecular route from wall deformation to intracellular signaling remains incompletely defined. Receptor-like kinases, wall-associated kinases, mechanosensitive channels, FERONIA-related pathways, RALF peptides, calcium influx, reactive oxygen species, and cytoskeletal remodeling have all been implicated in cell wall integrity sensing, but it is still unclear whether cells primarily detect altered stiffness, plasma membrane tension, wall-derived fragments, or combinations of these inputs. Closely related to this issue is how wall remodeling is integrated with broader hormonal and stress-signaling networks, including auxin, abscisic acid, ethylene, jasmonate, salicylic acid, calcium, and ROS pathways. Future work should therefore clarify which wall changes function as structural adaptations, which act as signaling cues, and how these processes are coordinated in specific developmental or stress contexts.

Another major limitation is our incomplete understanding of the wall as a spatially heterogeneous and multiscale structure. Plant cell walls are not uniform matrices; their composition and organization vary across cell types, developmental stages, and local domains such as growing zones, cell corners, pit fields, and infection sites. These local differences strongly influence extensibility, porosity, hydration, ion movement, and receptor accessibility, yet they are often obscured by bulk biochemical analyses. Resolving this complexity will require the integration of super-resolution imaging, atomic force microscopy, cryo-electron tomography, solid-state NMR, Raman-based methods, glycan probes, and spatial omics with direct mechanical measurements. Such approaches will be essential for linking nanoscale wall architecture to cell behavior and tissue-level function, and for understanding how local wall properties shape growth, transport, and defense.

The translational potential of cell wall research is substantial, but practical application depends on understanding trade-offs among growth, stress tolerance, and product quality. Wall traits influence lodging resistance, xylem function, pathogen defense, nutrient uptake, fruit firmness, seed quality, forage digestibility, and biomass processing efficiency. However, modifying one wall component often affects multiple traits simultaneously: reducing lignin may improve digestibility but weaken mechanical support, while altering pectin, suberin, or callose can enhance stress tolerance but also affect development or transport. Future crop improvement should therefore move beyond broad manipulation of wall polymers and instead aim for precise, tissue-specific, and context-dependent engineering of wall properties. Progress in this area will depend on combining genome editing, synthetic biology, single-cell and spatial omics, advanced imaging, biomechanical analysis, and computational modeling.

The relationships between cell wall composition, architecture, mechanics, signaling, and physiological responses are inherently complex and context-dependent. Rather than operating as a linear pathway, these processes form an interconnected regulatory network in which wall polymer composition and nanoscale organization shape local mechanical properties. These properties are sensed by receptors and mechanosensitive components at the plasma membrane, and the resulting signals are transduced through Ca^2+^, reactive oxygen species (ROS), and hormonal signaling networks to coordinate growth, stress adaptation, and defense. In turn, these responses feed back to regulate cell wall biosynthesis and remodeling. This integrated framework positions the cell wall as a dynamic regulatory interface that links extracellular mechanical cues to cellular and whole-plant responses. Ultimately, future cell wall research should shift from cataloging wall components to understanding the wall as a dynamic regulatory system that integrates mechanics, metabolism, development, and stress responses.

## Figures and Tables

**Table 1:** Structural features and biosynthetic enzymes of major plant hemicelluloses.

Hemicellulose Class	Backbone Architecture	Major Modifications	Representative Biosynthetic Enzymes	Subcellular Localization	References
Xyloglucan (XyG)	β-1,4-linked glucan	Xyl, Gal, Fuc, Ara, GalA	CslC; XXT1/2/5; MUR3; XLT2; XST1/XST2; XUT1; FUT1	Golgi	[41,42,43,44,45,46,47,48]
Xylan	β-1,4-linked xylan	GlcA/MeGlcA, Ara, acetyl, ferulate	IRX10/10L; IRX9/IRX9L; IRX14/IRX14L	Golgi	[38,39,40,49]
Mannan (±glucomannan)	β-1,4-linked mannan backbone	Gal, acetyl	CslA family; GMGT; MOATs	Golgi	[50,51,52,53]
Mixed-linkage glucan (MLG)	β-1,4/β-1,3-linked glucan	Usually unsubstituted	CslF; CslH	Golgi	[54,55,56,57]

**Table 2:** Putative cell wall integrity sensors in plants.

Receptor Family	Genes	CWI-Related Ligands	Role in CWI Signaling	Interaction Partners	Reference(s)
Catharanthus roseus RECEPTOR-LIKE KINASE1-LIKE (CrRLK1L) proteins	THESEUS1 (THE1)	Pectin and RALF34 peptide	Responses to cellulose biosynthesis inhibition and wall integrity surveillance	GEF4	[96,104,105]
	FERONIA (FER)	Pectin and RALF peptides	RALF receptor and scaffold in growth, immunity, and pectin-related wall sensing	LLG1	[98,106,107,108]
	ANXUR1 (ANX1), ANX2	RALF peptides	Maintaining pollen tube cell wall integrity	BUPS1/BUPS2, LLG2/LLG3	[109,110,111]
	ERULUS (ERU)	Unknown	Cell wall integrity maintenance in root hair	Unknown	[5]
	HERKULES1 (HERK1), HERK2	Unknown	Cell wall integrity maintenance and growth regulation	Unknown	[5]
Wall-associated kinases (WAKs)	WAK1, WAK2	Pectin and pectin-derived oligogalacturonides	Perceive pectin status and oligogalacturonides, linking wall perturbation to growth and immune responses	GRP-3, KAPP for WAK1	[5,102,103,112]
Leucine-rich repeat receptor-like kinases (LRR-RLKs)	FEI1, FEI2	Unknown	Responses to cellulose biosynthesis inhibition and regulate anisotropic growth	ACS5, ACS9	[5,113]
	MIK2	SCOOP family peptides	Responses to cellulose biosynthesis inhibition and cell wall stress	BAK1 and MDIS1	[114,115,116]
	STRUBBELIG (SUB)	Unknown	Required for cell wall integrity responses and developmental patterning	QKY; NHL3	[117,118,119,120]
Receptor-like protein	RLP12	Unknown	Responses to cellulose biosynthesis inhibition	Unknown	[121]
	PEPR1, PEPR2	Pep1-Pep6 for PEPR1; Pep1 and Pep2 for PEPR2	Modulate cell wall integrity responses and may dampen CWI signaling	BAK1, BIK1, and PBL1 for PEPR1; BAK1 for PEPR2	[5,122,123]

## Data Availability

No new data were created or analyzed in this study. Data sharing is not applicable to this article.

## References

[1] Somerville C., Bauer S., Brininstool G., Facette M., Hamann T., Milne J., Osborne E., Paredez A., Persson S., Raab T. (2004). Toward a systems approach to understanding plant cell walls. Science.

[2] Ragauskas A.J., Beckham G.T., Biddy M.J., Chandra R., Chen F., Davis M.F., Davison B.H., Dixon R.A., Gilna P., Keller M. (2014). Lignin valorization: Improving lignin processing in the biorefinery. Science.

[3] De Lorenzo G., Ferrari S., Giovannoni M., Mattei B., Cervone F. (2019). Cell wall traits that influence plant development, immunity, and bioconversion. Plant J..

[4] Dauphin B.G., Ranocha P., Dunand C., Burlat V. (2022). Cell-wall microdomain remodeling controls crucial developmental processes. Trends Plant Sci..

[5] Engelsdorf T., Gigli-Bisceglia N., Veerabagu M., McKenna J.F., Vaahtera L., Augstein F., Van der Does D., Zipfel C., Hamann T. (2018). The plant cell wall integrity maintenance and immune signaling systems cooperate to control stress responses in *Arabidopsis thaliana*. Sci. Signal..

[6] Cosgrove D.J. (2023). Structure and growth of plant cell walls. Nat. Rev. Mol. Cell Biol..

[7] Vaahtera L., Schulz J., Hamann T. (2019). Cell wall integrity maintenance during plant development and interaction with the environment. Nat. Plants.

[8] Zhong R., Ye Z.H. (2015). Secondary cell walls: Biosynthesis, patterned deposition and transcriptional regulation. Plant Cell Physiol..

[9] Nakano Y., Yamaguchi M., Endo H., Rejab N.A., Ohtani M. (2015). NAC-MYB-based transcriptional regulation of secondary cell wall biosynthesis in land plants. Front. Plant Sci..

[10] Lampugnani E.R., Khan G.A., Somssich M., Persson S. (2018). Building a plant cell wall at a glance. J. Cell Sci..

[11] Vogel J. (2008). Unique aspects of the grass cell wall. Curr. Opin. Plant Biol..

[12] Ušák D., Haluška S., Pleskot R. (2023). Callose synthesis at the center point of plant development-an evolutionary insight. Plant Physiol..

[13] Ma J.F., Yamaji N. (2006). Silicon uptake and accumulation in higher plants. Trends Plant Sci..

[14] Hepler P.K., Rounds C.M., Winship L.J. (2013). Control of cell wall extensibility during pollen tube growth. Mol. Plant.

[15] Rui Y., Anderson C.T. (2016). Functional analysis of cellulose and xyloglucan in the walls of stomatal guard cells of *Arabidopsis*. Plant Physiol..

[16] Grierson C., Nielsen E., Ketelaar T., Schiefelbein J. (2014). Root hairs. Arab. Book.

[17] Roberts J.A., Elliott K.A., Gonzalez-Carranza Z.H. (2002). Abscission, dehiscence, and other cell separation processes. Annu. Rev. Plant Biol..

[18] Arioli T., Peng L., Betzner A.S., Burn J., Wittke W., Herth W., Camilleri C., Hofte H., Plazinski J., Birch R. (1998). Molecular analysis of cellulose biosynthesis in *Arabidopsis*. Science.

[19] Taylor N.G., Howells R.M., Huttly A.K., Vickers K., Turner S.R. (2003). Interactions among three distinct CesA proteins essential for cellulose synthesis. Proc. Natl. Acad. Sci. USA.

[20] Paredez A.R., Somerville C.R., Ehrhardt D.W. (2006). Visualization of cellulose synthase demonstrates functional association with microtubules. Science.

[21] Richmond T.A., Somerville C.R. (2000). The cellulose synthase superfamily. Plant Physiol..

[22] Nicol F., His I., Jauneau A., Vernhettes S., Canut H., Höfte H. (1998). A plasma membrane-bound putative endo-1,4-β-D-glucanase is required for normal wall assembly and cell elongation in *Arabidopsis*. EMBO J..

[23] Vain T., Crowell E.F., Timpano H., Biot E., Desprez T., Mansoori N., Trindade L.M., Pagant S., Robert S., Hofte H. (2014). The cellulase KORRIGAN is part of the cellulose synthase complex. Plant Physiol..

[24] Lei L., Zhang T., Strasser R., Lee C.M., Gonneau M., Mach L., Vernhettes S., Kim S.H., Cosgrove D.J., Li S. (2014). The jiaoyao1 mutant is an allele of korrigan1 that abolishes endoglucanase activity and affects the organization of both cellulose microfibrils and microtubules in *Arabidopsis*. Plant Cell.

[25] Schindelman G., Morikami A., Jung J., Baskin T.I., Carpita N.C., Derbyshire P., McCann M.C., Benfey P.N. (2001). COBRA encodes a putative GPI-anchored protein, which is polarly localized and necessary for oriented cell expansion in *Arabidopsis*. Genes Dev..

[26] Roudier F., Fernandez A.G., Fujita M., Himmelspach R., Borner G.H.H., Schindelman G., Song S., Baskin T.I., Dupree P., Wasteneys G.O. (2005). COBRA, an *Arabidopsis* extracellular glycosyl-phosphatidyl inositol-anchored protein, specifically controls highly anisotropic expansion through its involvement in cellulose microfibril orientation. Plant Cell.

[27] Zhang Y., Nikolovski N., Sorieul M., Vellosillo T., McFarlane H.E., Dupree R., Kesten C., Schneider R., Driemeier C., Lathe R. (2016). Golgi-localized STELLO proteins regulate the assembly and trafficking of cellulose synthase complexes in *Arabidopsis*. Nat. Commun..

[28] Zhu X., Li S., Pan S., Xin X., Gu Y. (2018). CSI1, PATROL1, and exocyst complex cooperate in delivery of cellulose synthase complexes to the plasma membrane. Proc. Natl. Acad. Sci. USA.

[29] Mueller S.C., Brown R.M. (1980). Evidence for an intramembrane component associated with a cellulose microfibril-synthesizing complex in higher plants. J. Cell Biol..

[30] Morgan J.L., Strumillo J., Zimmer J. (2013). Crystallographic snapshot of cellulose synthesis and membrane translocation. Nature.

[31] Morgan J.L., McNamara J.T., Fischer M., Rich J., Chen H.M., Withers S.G., Zimmer J. (2016). Observing cellulose biosynthesis and membrane translocation in crystallo. Nature.

[32] Purushotham P., Ho R., Zimmer J. (2020). Architecture of a catalytically active homotrimeric plant cellulose synthase complex. Science.

[33] Fernandes A.N., Thomas L.H., Altaner C.M., Callow P., Forsyth V.T., Apperley D.C., Kennedy C.J., Jarvis M.C. (2011). Nanostructure of cellulose microfibrils in spruce wood. Proc. Natl. Acad. Sci. USA.

[34] Song B., Zhao S., Shen W., Collings C., Ding S.Y. (2020). Direct measurement of plant cellulose microfibril and bundles in native cell walls. Front. Plant Sci..

[35] Gu Y., Kaplinsky N., Bringmann M., Cobb A., Carroll A., Sampathkumar A., Baskin T.I., Persson S., Somerville C.R. (2010). Identification of a cellulose synthase-associated protein required for cellulose biosynthesis. Proc. Natl. Acad. Sci. USA.

[36] Endler A., Kesten C., Schneider R., Zhang Y., Ivakov A., Froehlich A., Funke N., Persson S. (2015). A mechanism for sustained cellulose synthesis during salt stress. Cell.

[37] Scheller H.V., Ulvskov P. (2010). Hemicelluloses. Annu. Rev. Plant Biol..

[38] Brown D.M., Goubet F., Wong V.W., Goodacre R., Stephens E., Dupree P., Turner S.R. (2007). Comparison of five xylan synthesis mutants reveals new insight into the mechanisms of xylan synthesis. Plant J..

[39] Brown D.M., Zhang Z., Stephens E., Dupree P., Turner S.R. (2009). Characterization of IRX10 and IRX10-like reveals an essential role in glucuronoxylan biosynthesis in *Arabidopsis*. Plant J..

[40] Wu A.M., Hornblad E., Voxeur A., Gerber L., Rihouey C., Lerouge P., Marchant A. (2010). Analysis of the *Arabidopsis* IRX9/IRX9-L and IRX14/IRX14-L pairs of glycosyltransferase genes reveals critical contributions to biosynthesis of the hemicellulose glucuronoxylan. Plant Physiol..

[41] Cocuron J.C., Lerouxel O., Drakakaki G., Alonso A.P., Liepman A.H., Keegstra K., Raikhel N., Wilkerson C.G. (2007). A gene from the cellulose synthase-like C family encodes a β-1,4 glucan synthase. Proc. Natl. Acad. Sci. USA.

[42] Kim S.J., Chandrasekar B., Rea A.C., Danhof L., Zemelis-Durfee S., Thrower N., Shepard Z.S., Pauly M., Brandizzi F., Keegstra K. (2020). The synthesis of xyloglucan, an abundant plant cell wall polysaccharide, requires CSLC function. Proc. Natl. Acad. Sci. USA.

[43] Vanzin G.F., Madson M., Carpita N.C., Raikhel N.V., Keegstra K., Reiter W.D. (2002). The mur2 mutant of *Arabidopsis thaliana* lacks fucosylated xyloglucan because of a lesion in fucosyltransferase AtFUT1. Proc. Natl. Acad. Sci. USA.

[44] Madson M., Dunand C., Li X., Verma R., Vanzin G.F., Caplan J., Shoue D.A., Carpita N.C., Reiter W.D. (2003). The MUR3 gene of *Arabidopsis* encodes a xyloglucan galactosyltransferase that is evolutionarily related to animal exostosins. Plant Cell.

[45] Cavalier D.M., Lerouxel O., Neumetzler L., Yamauchi K., Reinecke A., Freshour G., Zabotina O.A., Hahn M.G., Burgert I., Pauly M. (2008). Disrupting two *Arabidopsis thaliana* xylosyltransferase genes results in plants deficient in xyloglucan, a major primary cell wall component. Plant Cell.

[46] Jensen J.K., Schultink A., Keegstra K., Wilkerson C.G., Pauly M. (2012). RNA-seq analysis of developing nasturtium seeds [*Tropaeolum majus*]: Identification and characterization of an additional galactosyltransferase involved in xyloglucan biosynthesis. Mol. Plant.

[47] Peña M.J., Kong Y., York W.S., O’Neill M.A. (2012). A galacturonic acid-containing xyloglucan is involved in *Arabidopsis* root hair tip growth. Plant Cell.

[48] Schultink A., Cheng K., Park Y.B., Cosgrove D.J., Pauly M. (2013). The identification of two arabinosyltransferases from tomato reveals functional equivalency of xyloglucan side chain substituents. Plant Physiol..

[49] Urbanowicz B.R., Pena M.J., Moniz H.A., Moremen K.W., York W.S. (2014). Two *Arabidopsis* proteins synthesize acetylated xylan in vitro. Plant J..

[50] Dhugga K.S., Barreirro R., Whistten B., Stecca K., Hazebroek J., Randhawa G.S., Dolan M., Kinney A.J., Tomes D., Nichols S. (2004). Guar seed β-mannan synthase is a member of the cellulose synthase super gene family. Science.

[51] Voiniciuc C. (2022). Modern mannan: A hemicellulose’s journey. New Phytol..

[52] Edwards M.E., Choo T.S., Dickson C.A., Scott C., Gidley M.J., Reid J.S. (2004). The seeds of Lotus japonicus lines transformed with sense, antisense, and sense/antisense galactomannan galactosyltransferase constructs have structurally altered galactomannans in their endosperm cell walls. Plant Physiol..

[53] Zhong R.Q., Cui D.T., Ye Z.H. (2018). Members of the DUF231 family are O-acetyltransferases catalyzing 2-O- and 3-O-acetylation of mannan. Plant Cell Physiol..

[54] Burton R.A., Wilson S.M., Hrmova M., Harvey A.J., Shirley N.J., Medhurst A., Stone B.A., Newbigin E.J., Bacic A., Fincher G.B. (2006). Cellulose synthase-like CslF genes mediate the synthesis of cell wall [1,3;1,4]-β-D-glucans. Science.

[55] Vega-Sanchez M.E., Verhertbruggen Y., Christensen U., Chen X., Sharma V., Varanasi P., Jobling S.A., Talbot M., White R.G., Joo M. (2012). Loss of cellulose synthase-like F6 function affects mixed-linkage glucan deposition, cell wall mechanical properties, and defense responses in rice. Plant Physiol..

[56] Doblin M.S., Pettolino F.A., Wilson S.M., Campbell R., Burton R.A., Fincher G.B., Newbigin E., Bacic A. (2009). A barley cellulose synthase-like CSLH gene mediates [1,3;1,4]-β-D-glucan synthesis in transgenic *Arabidopsis*. Proc. Natl. Acad. Sci. USA.

[57] Wilson S.M., Ho Y.Y., Lampugnani E.R., Van de Meene A.M.L., Bain M.P., Bacic A., Doblin M.S. (2015). Determining the subcellular location of synthesis and assembly of the cell wall polysaccharide [1,3;1,4]-β-D-glucan in grasses. Plant Cell.

[58] Xiao C., Zhang T., Zheng Y., Cosgrove D.J., Anderson C.T. (2016). Xyloglucan deficiency disrupts microtubule stability and cellulose biosynthesis in *Arabidopsis*, altering cell growth and morphogenesis. Plant Physiol..

[59] Lee C., Zhong R., Richardson E., Himmelsbach D., Mcphail B., Ye Z. (2007). The PARVUS gene is expressed in cells undergoing secondary wall thickening and is essential for glucuronoxylan biosynthesis. Plant Cell Physiol..

[60] Peña M., Zhong R., Zhou G.K., Richardson E., O’Neill M., Darvill A., York W., Ye Z. (2007). *Arabidopsis* irregular xylem8 and irregular xylem9: Implications for the complexity of glucuronoxylan biosynthesis. Plant Cell.

[61] Caffall K.H., Mohnen D. (2009). The structure, function, and biosynthesis of plant cell wall pectic polysaccharides. Carbohydr. Res..

[62] Amos R.A., Pattathil S., Yang J.Y., Atmodjo M.A., Urbanowicz B.R., Moremen K.W., Mohnen D. (2018). A two-phase model for the non-processive biosynthesis of homogalacturonan polysaccharides by the GAUT1:GAUT7 complex. J. Biol. Chem..

[63] Engle K.A., Amos R.A., Yang J.Y., Glushka J., Atmodjo M.A., Tan L., Huang C., Moremen K.W., Mohnen D. (2022). Multiple *Arabidopsis* galacturonosyltransferases synthesize polymeric homogalacturonan by oligosaccharide acceptor-dependent or de novo synthesis. Plant J..

[64] Takenaka Y., Kato K., Ogawa-Ohnishi M., Tsuruhama K., Kajiura H., Yagyu K., Takeda A., Takeda Y., Kunieda T., Hara-Nishimura I. (2018). Pectin RG-I rhamnosyltransferases represent a novel plant-specific glycosyltransferase family. Nat. Plants.

[65] Wolf S., Mouille G., Pelloux J. (2009). Homogalacturonan methyl-esterification and plant development. Mol. Plant.

[66] Braccini I., Pérez S. (2001). Molecular basis of Ca^2+^-induced gelation in alginates and pectins: The egg-box model revisited. Biomacromolecules.

[67] Ralph J., Lapierre C., Boerjan W. (2019). Lignin structure and its engineering. Curr. Opin. Biotechnol..

[68] Humphreys J.M., Hemm M.R., Chapple C. (1999). New routes for lignin biosynthesis defined by biochemical characterization of recombinant ferulate 5-hydroxylase, a multifunctional cytochrome P450-dependent monooxygenase. Proc. Natl. Acad. Sci. USA.

[69] Vanholme R., Cesarino I., Rataj K., Xiao Y., Sundin L., Goeminne G., Kim H., Cross J., Morreel K., Ralph J. (2013). Caffeoyl shikimate esterase [CSE] is an enzyme in the lignin biosynthetic pathway in *Arabidopsis*. Science.

[70] Barros J., Shrestha H.K., Serrani-Yarce J.C., Engle N., Abraham P.E., Tschaplinski T.J., Hettich R.L., Dixon R.A. (2022). Proteomic and metabolic disturbances in lignin modified *Brachypodium distachyon*. Plant Cell.

[71] Boerjan W., Ralph J., Baucher M. (2003). Lignin biosynthesis. Annu. Rev. Plant Biol..

[72] Oyarce P., De Meester B., Fonseca F., de Vries L., Goeminne G., Pallidis A., De Rycke R., Tsuji Y., Li Y., Van den Bosch S. (2019). Introducing curcumin biosynthesis in *Arabidopsis* enhances lignocellulosic biomass processing. Nat. Plants.

[73] Mottiar Y., Karlen S.D., Goacher R.E., Ralph J., Mansfield S.D. (2023). Metabolic engineering of p-hydroxybenzoate in poplar lignin. Plant Biotechnol. J..

[74] van Parijs F.R.D., Morreel K., Ralph J., Boerjan W., Merks R.M.H. (2010). Modeling lignin polymerization. I. Simulation model of dehydrogenation polymers. Plant Physiol..

[75] Bao W., O’Malley D.M., Whetten R., Sederoff R.R. (1993). A laccase associated with lignification in loblolly pine xylem. Science.

[76] Zhao Q., Nakashima J., Chen F., Yin Y., Fu C., Yun J., Shao H., Wang X., Wang Z.Y., Dixon R.A. (2013). LACCASE is necessary and non-redundant with PEROXIDASE for lignin polymerization during vascular development in *Arabidopsis thaliana*. Plant Cell.

[77] Lee Y., Rubio M.C., Alassimone J., Geldner N. (2013). A mechanism for localized lignin deposition in the endodermis. Cell.

[78] Zhang J., Liu Y., Li C., Yin B., Liu X., Guo X., Zhang C., Liu D., Hwang I., Li H. (2022). PtomtAPX is an autonomous lignification peroxidase during the earliest stage of secondary wall formation in *Populus tomentosa* carr. Nat. Plants.

[79] Chou E.Y., Schuetz M., Hoffmann N., Watanabe Y., Sibout R., Samuels A.L. (2018). Distribution, mobility, and anchoring of lignin-related oxidative enzymes in *Arabidopsis* secondary cell walls. J. Exp. Bot..

[80] Barbosa I.C.R., Rojas-Murcia N., Geldner N. (2019). The Casparian strip-one ring to bring cell biology to lignification?. Curr. Opin. Biotechnol..

[81] Marcuello C., Bercu N., Foulon L., Chabbert B., Molinari M., Aguié-Béghin V. (2025). Impact of lignin structuration on its chemical andadhesive properties at the nanoscale: Involvement with native and polymer matrices. Nanoscale.

[82] Klenovšek U., Vrabič-Brodnjak U. (2026). Lignin-based adhesives for various packaging applications. Polymers.

[83] Keegstra K., Talmadge K.W., Bauer W.D., Albersheim P. (1973). The structure of plant walls III. A model of the walls of suspension-cultured sycamore cells. Plant Physiol..

[84] Kang X., Kirui A., Dickwella Widanage M.C., Mentick-Vigier F., Cosgrove D.J., Wang T. (2019). Lignin-polysaccharide interactions in plant secondary cell walls revealed by solid-state NMR. Nat. Commun..

[85] Zhang L., Gao C., Mentink-Vigier F., Tang L., Zhang D., Wang S., Cao S., Xu Z., Liu X., Wang T. (2019). Arabinosyl deacetylase modulates the arabinoxylan acetylation profile and secondary wall formation. Plant Cell.

[86] Liu L., Shang-Guan K., Zhang B., Liu X., Yan M., Zhang L., Shi Y., Zhang M., Qian Q., Li J. (2013). Brittle Culm1, a COBRA-like protein, functions in cellulose assembly through binding cellulose microfibrils. PLoS Genet..

[87] Ganguly A., Zhu C., Chen W., Dixit R. (2020). FRA1 kinesin modulates the lateral stability of cortical microtubules through cellulose synthase-microtubule uncoupling proteins. Plant Cell.

[88] Du J., Kirui A., Huang S., Wang L., Barnes W.J., Kiemle S., Zheng Y., Rui Y., Ruan M., Qi S. (2020). Mutations in the pectin methyltransferase QUASIMODO2 influence cellulose biosynthesis and wall integrity in *Arabidopsis thaliana*. Plant Cell.

[89] Simmons T.J., Mortimer J.C., Bernardinelli O.D., Poppler A.C., Brown S.P., deAzevedo E.R., Dupree R., Dupree P. (2016). Folding of xylan onto cellulose fibrils in plant cell walls revealed by solid-state NMR. Nat. Commun..

[90] Zhang T., Vavylonis D., Durachko D.M., Cosgrove D.J. (2017). Nanoscale movements of cellulose microfibrils in primary cell walls. Nat. Plants.

[91] Hamann T. (2015). The plant cell wall integrity maintenance mechanism-concepts for organization and mode of action. Plant Cell Physiol..

[92] Ha C.M., Rao X., Saxena G., Dixon R.A. (2021). Growth–defense trade-offs and yield loss in plants with engineered cell walls. New Phytol..

[93] Shedletzky E., Shmuel M., Delmer D.P., Lamport D.T.A. (1990). Adaptation and growth of tomato cells on the herbicide 2,6-dichlorobenzonitrile leads to production of unique cell walls virtually lacking a cellulose-xyloglucan network. Plant Physiol..

[94] Shedletzky E., Shmuel M., Trainin T., Kalman S., Delmer D. (1992). Cell wall structure in cells adapted to growth on the cellulose-synthesis inhibitor 2,6-dichlorobenzonitrile: A comparison between two dicotyledonous plants and a graminaceous monocot. Plant Physiol..

[95] Manfield I.W., Orfila C., Mccartney L., Harholt J., Bernal A.J., Scheller H.V., Gilmartin P.M., Mikkelsen J.D., Paul Knox J., Willats W.G. (2004). Novel cell wall architecture of isoxaben-habituated *Arabidopsis* suspension-cultured cells: Global transcript profiling and cellular analysis. Plant J..

[96] Hematy K., Sado P.E., Van Tuinen A., Rochange S., Desnos T., Balzergue S., Pelletier S., Renou J.P., Höfte H. (2007). A receptor-like kinase mediates the response of *Arabidopsis* cells to the inhibition of cellulose synthesis. Curr. Biol..

[97] Davidsson P., Broberg M., Kariola T., Sipari N., Pirhonen M., Palva E.T. (2017). Short oligogalacturonides induce pathogen resistance-associated gene expression in *Arabidopsis thaliana*. BMC Plant Biol..

[98] Souza C.A., Li S., Lin A.Z., Boutrot F., Grossmann G., Zipfel C., Somerville S.C. (2017). Cellulose-derived oligomers act as damage-associated molecular patterns and trigger defense-like responses. Plant Physiol..

[99] Molina A., Jorda L., Torres M.A., Martin-Dacal M., Berlanga D.J., Fernandez-Calvo P., Gomez-Rubio E., Martin-Santamaria S. (2024). Plant cell wall-mediated disease resistance: Current understanding and future perspectives. Mol. Plant.

[100] Haruta M., Sabat G., Stecker K., Minkoff B.B., Sussman M.R. (2014). A peptide hormone and its receptor protein kinase regulate plant cell expansion. Science.

[101] Yamaguchi Y., Pearce G., Ryan C.A. (2006). The cell surface leucine-rich repeat receptor for AtPep1, an endogenous peptide elicitor in *Arabidopsis*, is functional in transgenic tobacco cells. Proc. Natl. Acad. Sci. USA.

[102] Brutus A., Sicilia F., Macone A., Cervone F., De Lorenzo G. (2010). A domain swap approach reveals a role of the plant wall-associated kinase 1 (WAK1) as a receptor of oligogalacturonides. Proc. Natl. Acad. Sci. USA.

[103] Kohorn B.D., Kohorn S.L. (2012). The cell wall-associated kinases, WAKs, as pectin receptors. Front. Plant Sci..

[104] Gonneau M., Desprez T., Martin M., Doblas V.G., Bacete L., Miart F., Sormani R., Hématy K., Renou J., Landrein B. (2018). Receptor kinase THESEUS1 is a rapid alkalinization factor 34 receptor in *Arabidopsis*. Curr. Biol..

[105] Qu S., Zhang X., Song Y., Lin J., Shan X. (2017). THESEUS1 positively modulates plant defense responses against *Botrytis cinerea* through GUANINE EXCHANGE FACTOR4 signaling. J. Integr. Plant Biol..

[106] Feng W., Kita D., Peaucelle A., Cartwright H.N., Doan V., Duan Q., Liu M.C., Maman J., Steinhorst L., Schmitz-Thom I. (2018). The FERONIA receptor kinase maintains cell-wall integrity during salt stress through Ca^2+^ signaling. Curr. Biol..

[107] Stegmann M., Monaghan J., Smakowska-Luzan E., Rovenich H., Lehner A., Holton N., Belkhadir Y., Zipfel C. (2017). The receptor kinase FER is a RALF-regulated scaffold controlling plant immune signaling. Science.

[108] Li C., Yeh F.L., Cheung A.Y., Duan Q., Kita D., Liu M.C., Maman J., Luu E.J., Wu B.W., Gates L. (2015). Glycosylphosphatidylinositol-anchored proteins as chaperones and co-receptors for FERONIA receptor kinase signaling in *Arabidopsis*. eLife.

[109] Boisson-Dernier A., Lituiev D.S., Nestorova A., Franck C.M., Thirugnanarajah S., Grossniklaus U. (2013). ANXUR receptor-like kinases coordinate cell wall integrity with growth at the pollen tube tip via NADPH oxidases. PLoS Biol..

[110] Ge Z., Bergonci T., Zhao Y., Zou Y., Du S., Liu M.-C., Luo X., Ruan H., Garcia-Valencia L.E., Zhong S. (2017). *Arabidopsis* pollen tube integrity and sperm release are regulated by RALF-mediated signaling. Science.

[111] Ge Z., Zhao Y., Liu M.-C., Zhou L.-Z., Wang L., Zhong S., Hou S., Jiang J., Liu T., Huang Q. (2019). LLG2/3 are co-receptors in BUPS/ANX-RALF signaling to regulate *Arabidopsis* pollen tube integrity. Curr. Biol..

[112] Gramegna G., Modesti V., Savatin D.V., Sicilia F., Cervone F., De Lorenzo G. (2016). GRP-3 and KAPP, encoding interactors of WAK1, negatively affect defense responses induced by oligogalacturonides and local response to wounding. J. Exp. Bot..

[113] Xu S.L., Rahman A., Baskin T.I., Kieber J.J. (2008). Two leucine-rich repeat receptor kinases mediate signaling, linking cell wall biosynthesis and ACC synthase in *Arabidopsis*. Plant Cell.

[114] Van der Does D., Boutrot F., Engelsdorf T., Rhodes J., McKenna J.F., Vernhettes S., Koevoets I., Tintor N., Veerabagu M., Miedes E. (2017). The *Arabidopsis* leucine-rich repeat receptor kinase MIK2/LRR-KISS connects cell wall integrity sensing, root growth and response to abiotic and biotic stresses. PLoS Genet..

[115] Rhodes J., Yang H., Moussu S., Boutrot F., Santiago J., Zipfel C. (2021). Perception of a divergent family of phytocytokines by the *Arabidopsis* receptor kinase MIK2. Nat. Commun..

[116] Wang T., Liang L., Xue Y., Jia P.F., Chen W., Zhang M.X., Wang Y.C., Li H.J., Yang W.C. (2016). A receptor heteromer mediates the male perception of female attractants in plants. Nature.

[117] Chaudhary A., Chen X., Leśniewska B., Boikine R., Gao J., Wolf S., Schneitz K. (2021). Cell wall damage attenuates root hair patterning and tissue morphogenesis mediated by the receptor kinase STRUBBELIG. Development.

[118] Vaddepalli P., Herrmann A., Fulton L., Oelschner M., Hillmer S., Stratil T.F., Fastner A., Hammes U.Z., Ott T., Robinson D.G. (2014). The C2-domain protein QUIRKY and the receptor-like kinase STRUBBELIG localize to plasmodesmata and mediate tissue morphogenesis in *Arabidopsis thaliana*. Development.

[119] Chen X., Leśniewska B., Boikine R., Yun N., Mody T.A., Vaddepalli P., Schneitz K. (2023). *Arabidopsis* MCTP family member QUIRKY regulates the formation of the STRUBBELIG receptor kinase complex. Plant Physiol..

[120] Boikine R., Chaudhary A., Mergner J., Leicher H., Stegmann M., Kuster B., Schneitz K. (2026). A STRUBBELIG-NHL3 cell surface receptor complex mediates the response to cellulose deficiency in *Arabidopsis*. Nat. Plants.

[121] Bacete L., Schulz J., Engelsdorf T., Bartosova Z., Vaahtera L., Yan G., Gerhold J.M., Tichá T., Øvstebø C., Gigli-Bisceglia N. (2022). THESEUS1 modulates cell wall stiffness and abscisic acid production in *Arabidopsis thaliana*. Proc. Natl. Acad. Sci. USA.

[122] Liu Z., Wu Y., Yang F., Zhang Y., Chen S., Xie Q., Tian X., Zhou J.-M. (2013). BIK1 interacts with PEPRs to mediate ethylene-induced immunity. Proc. Natl. Acad. Sci. USA.

[123] Schulze B., Mentzel T., Jehle A.K., Mueller K., Beeler S., Boller T., Felix G., Chinchilla D. (2010). Rapid heteromerization and phosphorylation of ligand-activated plant transmembrane receptors and their associated kinase BAK1. J. Biol. Chem..

[124] Hoermayer L., Montesinos J.C., Marhava P., Benková E., Yoshida S., Friml J. (2020). Wounding-induced changes in cellular pressure and localized auxin signalling spatially coordinate restorative divisions in roots. Proc. Natl. Acad. Sci. USA.

[125] Canher B., Lanssens F., Zhang A., Bisht A., Mazumdar S., Heyman J., Wolf S., Melnyk C.W., De Veylder L. (2022). The regeneration factors ERF114 and ERF115 regulate auxin-mediated lateral root development in response to mechanical cues. Mol. Plant.

[126] Liu C., Yu H., Rao X., Li L., Dixon R.A. (2021). Abscisic acid regulates secondary cell-wall formation and lignin deposition in *Arabidopsis thaliana* through phosphorylation of NST1. Proc. Natl. Acad. Sci. USA.

[127] Xu Y., Du J., Hao R., Ma S., Ma Y., Zhou G., Hu R., Li S. (2025). Gibberellin signaling regulates pectin biosynthesis in *Arabidopsis*. Nat. Commun..

[128] Mielke S., Zimmer M., Meena M., Dreos R., Stellmach H., Hause B., Voiniciuc C., Gasperini D. (2021). Jasmonate biosynthesis arising from altered cell walls is prompted by turgor-driven mechanical compression. Sci. Adv..

[129] Jia H., Wang X., Wei T., Wang M., Liu X., Hua L., Ren X., Guo J., Li J. (2021). Exogenous salicylic acid regulates cell wall polysaccharides synthesis and pectin methylation to reduce Cd accumulation of tomato. Ecotoxicol. Environ. Saf..

[130] Zhang J., Liu Z., Farrar E., Li M., Lu H., Qu Z., Chara O., Mitsuda N., Sakamoto S., Xue F. (2026). Ethylene modulates cell wall mechanics for root responses to compaction. Nature.

[131] Burk D.H., Liu B., Zhong R., Morrison W.H., Ye Z.H. (2001). A katanin-like protein regulates normal cell wall biosynthesis and cell elongation. Plant Cell.

[132] Zhang B., Deng L., Qian Q., Xiong G., Zeng D., Li R., Guo L., Li J., Zhou Y. (2009). A missense mutation in the transmembrane domain of CESA4 affects protein abundance in the plasma membrane and results in abnormal cell wall biosynthesis in rice. Plant Mol. Biol..

[133] Zhang B., Zhang L., Li F., Zhang D., Liu X., Wang H., Xu Z., Chu C., Zhou Y. (2017). Control of secondary cell wall patterning involves xylan deacetylation by a GDSL esterase. Nat. Plants.

[134] Jones L., Ennos A.R., Turner S.R. (2001). Cloning and characterization of irregular xylem4 (irx4): A severely lignin-deficient mutant of *Arabidopsis*. Plant J..

[135] Crowe J.D., Hao P., Pattathil S., Pan H., Ding S.Y., Hodge D.B., Jensen J.K. (2021). Xylan is critical for proper bundling and alignment of cellulose microfibrils in plant secondary cell walls. Front. Plant Sci..

[136] Wang H., Yang H., Wen Z., Gao C., Gao Y., Tian Y., Xu Z., Liu X., Persson S., Zhang B. (2022). Xylan-based nanocompartments orchestrate plant vessel wall patterning. Nat. Plants.

[137] Oda Y., Iida Y., Kondo Y., Fukuda H. (2010). Wood cell-wall structure requires local 2D-microtubule disassembly by a novel plasma membrane-anchored protein. Curr. Biol..

[138] Oda Y., Fukuda H. (2013). Rho of plant GTPase signaling regulates the behavior of *Arabidopsis* kinesin-13A to establish secondary cell wall patterns. Plant Cell.

[139] Watanabe Y., Meents M.J., McDonnell L.M., Barkwill S., Sampathkumar A., Cartwright H.N., Demura T., Ehrhardt D.W., Samuels A.L., Mansfield S.D. (2015). Visualization of cellulose synthases in *Arabidopsis* secondary cell walls. Science.

[140] Zhang L., Gao Y., Xu Z., Hu J., Wen Z., Li J., Gao C., Zhou Y., Zhang B. (2025). Shaping pit structure in vessel walls sustains xylem hydraulics and grain yield. Cell.

[141] Rui Y., Xiao C., Yi H., Kandemir B., Wang J.Z., Puri V.M., Anderson C.T. (2017). POLYGALACTURONASE INVOLVED IN EXPANSION3 Functions in Seedling Development, Rosette Growth, and Stomatal Dynamics in *Arabidopsis thaliana*. Plant Cell.

[142] Wachsman G., Zhang J., Moreno-Risueno M.A., Anderson C.T., Benfey P.N. (2020). Cell wall remodeling and vesicle trafficking mediate the root clock in *Arabidopsis*. Science.

[143] Savaldi-Goldstein S., Peto C., Chory J. (2007). The epidermis both drives and restricts plant shoot growth. Nature.

[144] Zhu X., Chen X., Liu Y., Zhu Y., Gao G., Lan M., Fu Y., Gu Y., Han H., Cai W. (2025). Cell wall patterning regulates plant stem cell dynamics. Science.

[145] Jiang L., Yang S.L., Xie L.F., Puah C.S., Zhang X.Q., Yang W.C., Sundaresan V., Ye D. (2005). *VANGUARD1* encodes a pectin methylesterase that enhances pollen tube growth in the *Arabidopsis* style and transmitting tract. Plant Cell.

[146] Zhang Z., Zhang B., Chen Z., Zhang D., Zhang H., Wang H., Zhang Y., Cai D., Liu J., Xiao S. (2018). A PECTIN METHYLESTERASE gene at the maize Ga1 locus confers male function in unilateral cross-incompatibility. Nat. Commun..

[147] Haas K.T., Wightman R., Meyerowitz E.M., Peaucelle A. (2020). Pectin homogalacturonan nanofilament expansion drives morphogenesis in plant epidermal cells. Science.

[148] Chen Z., Hong X., Zhang H., Wang Y., Li X., Zhu J.K., Gong Z. (2005). Disruption of the cellulose synthase gene, AtCesA8/IRX1, enhances drought and osmotic stress tolerance in *Arabidopsis*. Plant J..

[149] Chen Y., Zhang B., Li C., Lei C., Kong C., Yang Y., Gong M. (2019). A comprehensive expression analysis of the expansin gene family in potato (*Solanum tuberosum*) discloses stress-responsive expansin-like B genes for drought and heat tolerances. PLoS ONE.

[150] Gao Y.Q., Su Y., Chao D.Y. (2024). Exploring the function of plant root diffusion barriers in sealing and shielding for environmental adaptation. Nat. Plants.

[151] Tenhaken R. (2014). Cell wall remodeling under abiotic stress. Front. Plant Sci..

[152] Yan J., He H., Fang L., Zhang A. (2018). Pectin methylesterase31 positively regulates salt stress tolerance in *Arabidopsis*. Biochem. Biophys. Res. Commun..

[153] Zhou T., Wu P., Chen J., Du X., Feng Y., Hua Y. (2024). Pectin demethylation-mediated cell wall Na^+^ retention positively regulates salt stress tolerance in oilseed rape. Theor. Appl. Genet..

[154] Fujita S., Pytela J., Hotta T., Kato T., Hamada T., Akamatsu R., Ishida Y., Kutsuna N., Hasezawa S., Nomura Y. (2013). An atypical tubulin kinase mediates stress-induced microtubule depolymerization in *Arabidopsis*. Curr. Biol..

[155] Wang Y., Cao Y., Liang X., Zhuang J., Wang X., Qin F., Jiang C. (2022). A dirigent family protein confers variation of Casparian strip thickness and salt tolerance in maize. Nat. Commun..

[156] Qu T., Liu R., Wang W., An L., Chen T., Liu G., Zhao Z. (2011). Brassinosteroids regulate pectin methylesterase activity and AtPME41 expression in *Arabidopsis* under chilling stress. Cryobiology.

[157] Huang Y.C., Wu H.C., Wang Y.D., Liu C.H., Lin C.C., Luo D.L., Jinn T.L. (2017). PECTIN METHYLESTERASE34 contributes to heat tolerance through its role in promoting stomatal movement. Plant Physiol..

[158] Takahashi D., Johnson K.L., Hao P., Tuong T., Erban A., Sampathkumar A., Bacic A., Livingston D.P., Kopka J., Kuroha T. (2021). Cell wall modification by the xyloglucan endotransglucosylase/hydrolase XTH19 influences freezing tolerance after cold and sub-zero acclimation. Plant Cell Environ..

[159] Xiao J., Sui X., Xu Z., Liang L., Tang W., Tang Y., Sun B., Lai Y., Huang Z., Zheng Y. (2025). CaNAC76 enhances lignin content and cold resistance in pepper by regulating CaCAD1. Int. J. Biol. Macromol..

[160] De Caroli M., Manno E., Piro G., Lenucci M.S. (2021). Ride to cell wall: *Arabidopsis* XTH11, XTH29 and XTH33 exhibit different secretion pathways and responses to heat and drought stress. Plant J..

[161] Wang X., Ma J., He F., Wang L., Zhang T., Liu D., Xu Y., Li F., Feng X. (2024). A study on the functional identification of overexpressing winter wheat expansin gene TaEXPA7-B in rice under salt stress. Int. J. Mol. Sci..

[162] Tanaka K., Heil M. (2021). Damage-Associated Molecular Patterns (DAMPs) in Plant Innate Immunity: Applying the Danger Model and Evolutionary Perspectives. Annu. Rev. Phytopathol..

[163] Gallego-Giraldo L., Pose-Albacete S., Pattathil S., Peralta A.G., Hahn M., Ayre B.G., Sunuwar H., Hernandez J., Patel M., Shah J. (2018). Elicitors and defense gene induction in plants with altered lignin compositions. New Phytol..

